# Pan-cancer analysis of genomic scar signatures associated with homologous recombination deficiency suggests novel indications for existing cancer drugs

**DOI:** 10.1186/s40364-015-0033-4

**Published:** 2015-05-01

**Authors:** Andrea M Marquard, Aron C Eklund, Tejal Joshi, Marcin Krzystanek, Francesco Favero, Zhigang C Wang, Andrea L Richardson, Daniel P Silver, Zoltan Szallasi, Nicolai J Birkbak

**Affiliations:** Center for Biological Sequence Analysis, Department of Systems Biology, Technical University of Denmark, Kemitorvet 8, 2800 Lyngby, Denmark; Department of Cancer Biology, Dana-Farber Cancer Institute, 450 Brookline Avenue, 02215 Boston, Massachusetts USA; Department of Surgery, Brigham and Women’s Hospital, 75 Francis Street, 02115 Boston, Massachusetts USA; Department of Pathology, Brigham and Women’s Hospital, 75 Francis Street, 02115 Boston, Massachusetts USA; Department of Medical Oncology, Dana-Farber Cancer Institute, 450 Brookline Avenue, 02215 Boston, Massachusetts USA; Harvard Medical School, Children’s Hospital Informatics Program at the Harvard-Massachusetts Institute of Technology Division of Health Sciences and Technology (CHIP@HST), 320 Longwood Avenue, Boston, Massachusetts USA

**Keywords:** Cancer, Homologous recombination deficiency, Genomic scars

## Abstract

**Background:**

Ovarian and triple-negative breast cancers with BRCA1 or BRCA2 loss are highly sensitive to treatment with PARP inhibitors and platinum-based cytotoxic agents and show an accumulation of genomic scars in the form of gross DNA copy number aberrations. Cancers without BRCA1 or BRCA2 loss but with accumulation of similar genomic scars also show increased sensitivity to platinum-based chemotherapy. Therefore, reliable biomarkers to identify DNA repair-deficient cancers prior to treatment may be useful for directing patients to platinum chemotherapy and possibly PARP inhibitors. Recently, three SNP array-based signatures of chromosomal instability were published that each quantitate a distinct type of genomic scar considered likely to be caused by improper DNA repair. They measure telomeric allelic imbalance (named NtAI), large scale transition (named LST), and loss of heterozygosity (named HRD-LOH), and it is suggested that these signatures may act as biomarkers for the state of DNA repair deficiency in a given cancer.

**Results:**

We explored the pan-cancer distribution of scores of the three signatures utilizing a panel of 5371 tumors representing 15 cancer types from The Cancer Genome Atlas, and found a good correlation between scores of the three signatures (Spearman’s ρ 0.73–0.87). In addition we found that cancer types ordinarily receiving platinum as standard of care have higher median scores of all three signatures. Interestingly, we also found that smaller subpopulations of high-scoring tumors exist in most cancer types, including those for which platinum chemotherapy is not standard therapy.

**Conclusions:**

Within several cancer types that are not ordinarily treated with platinum chemotherapy, we identified tumors with high levels of the three genomic biomarkers. These tumors represent identifiable subtypes of patients which may be strong candidates for clinical trials with PARP inhibitors or platinum-based chemotherapeutic regimens.

**Electronic supplementary material:**

The online version of this article (doi:10.1186/s40364-015-0033-4) contains supplementary material, which is available to authorized users.

## Background

Personalized medicine in cancer aims to improve treatment outcome by matching the specific biological characteristics of a tumor with the most appropriate therapeutic option. One such biological characteristic is a defect in one of the DNA repair systems, which often leads to the accumulation of genomic damage, such as point mutations and short indels, as well as gross copy number aberrations, termed genomic scars, that may be gain or loss of large chromosomal regions, or even whole chromosomes [1,2]. The ability to repair DNA is a fundamental requirement to sustain cellular life, and thus multiple partially redundant repair pathways have evolved [3]. DNA repair defects are one determinant of therapeutic response to many chemotherapeutic agents. Depending on the type of DNA repair defects present, cancers may show increased sensitivity to treatment with certain DNA-damaging agents or drugs that interfere with other parts of the DNA repair system. This has been shown in particular with BRCA1 and BRCA2, key genes in both the homologous recombination (HR) and Fanconi Anemia (FA) pathways [4-6]. Ovarian and breast tumors with loss of either BRCA1 or BRCA2 are particularly sensitive to treatment with platinum-based DNA crosslinking agents [7-10]. In addition, loss of BRCA1/2 also causes sensitivity to inhibition of PARP1, a key gene in the base excision repair pathway and the target of several inhibitors currently undergoing clinical evaluation [3,11,12]. However, some tumors with no apparent loss of BRCA1 or BRCA2 exhibit similar patterns of genomic scars, and also show increased sensitivity to treatment with platinum drugs [8,13,14]. This suggests that defects in DNA repair other than BRCA1 or BRCA2 loss may also confer drug sensitivities similar to BRCA1/2 loss. As the patterns of genomic scars may be DNA repair pathway-specific rather than gene-specific, drug response signatures based on genomic scars may be reliable biomarkers for DNA repair deficiency and could be used to identify patients that would benefit from specific types of anti-cancer therapy [15].

**Figure 1:**
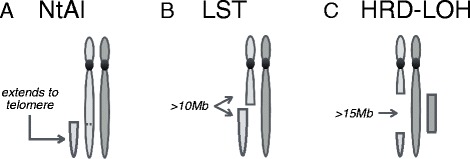
Overview of the type of genomic scars measured by each HR signature. Dark and light grey are used to indicate paternal and maternal chromosomes. **A**: Number of telomeric allelic imbalances (NtAI) counts the number of subtelomeric regions with allelic imbalance, that start beyond the centromere and extend to the telomere. **B**: Large-scale state transitions (LST) counts the number of chromosomal breaks between adjacent regions of at least 10 Mb. **C**: Homologous recombination deficiency score (HRD-LOH) measures the number of regions with LOH which are larger than 15 Mb, but shorter than the whole chromosome.

Three signatures, each measuring a specific type of genomic scar in SNP array data, were published in 2012 by three groups, including ours. The **N**umber of **t**elomeric **A**llelic **I**mbalances (NtAI), published by us, was based on a type of genomic scar accumulation which could predict response to platinum-based chemotherapy in triple negative breast cancer (TNBC, expressing neither estrogen, progesterone, nor HER2 receptors) and high grade serous ovarian cancer [8]. **L**arge **S**cale **T**ransition (LST), published by Popova et al., was based on a type of genomic scar which was associated with loss of BRCA1 or BRCA2 in TNBC [13]. The **H**omologous **R**ecombination **D**eficiency score (HRD-LOH^a^), published by Abkevich et al., was based on a type of genomic scar that was enriched in high grade serous ovarian cancer patients with loss of BRCA1 or BRCA2 [14]. The genomic scars measured by each signature are defined differently, although it is possible for a scar to be included in more than one signature (Figure 1). All three signatures are based on the assumption that summary measures of a defined type of genomic scar is proportional to the number of times an established cancer experienced error-prone DNA repair of a given type, which resulted in the measured genomic scar.

As these signatures measure similar genomic features and have been designed for the same purpose of guiding precision medicine, we here use The Cancer Genome Atlas (TCGA) to determine the distribution of the three signatures of genomic scars across 15 different cancer types. We find that cases with high levels of each signature can be found in 12 of the 15 cancer types, suggesting that it may be possible to use genomic scar signatures to identify patients with DNA repair deficiency predictive of platinum chemotherapy sensitivity prior to initiating therapy. We also investigate the association between high levels of each signature and increased ploidy, p53 loss, and other measures of genomic instability.

## Results

### Distribution of signature scores across cancer types

**Figure 2:**
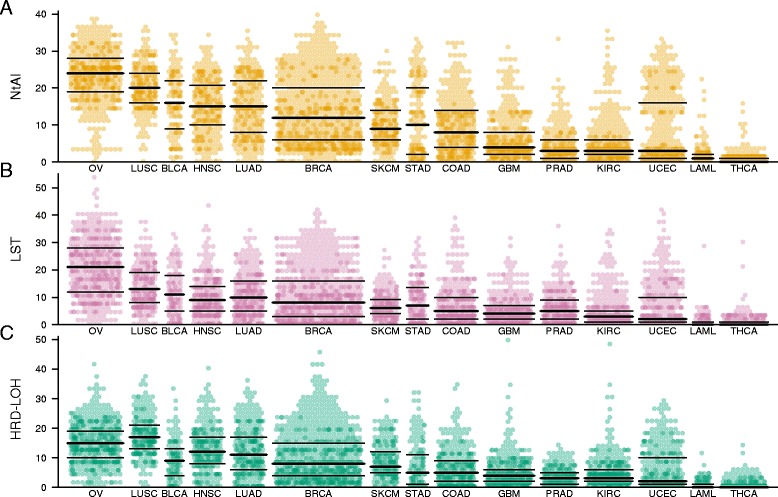
Distribution of signature scores across 15 cancer types. **A**: Distribution of NtAI scores. **B**: Distribution of LST scores. **C**: Distribution of HRD-LOH scores. Horizontal black lines indicate 25^th^, 50^th^ and 75^th^ percentiles.

We first determined the distribution of the scores of each signature in the 15 cancer types (Figure 2, Table 1). We observed a considerable variation in scores within each cancer type, ranging from low to high levels of all signature scores in most cancer types. This shows that the type of chromosomal aberrations that are counted by each signature is not restricted to a single or a few types of cancer, but rather represents a general aberration pattern that can be found across most cancer types. The exceptions were acute myeloid leukemia (AML) and thyroid cancer, which predominantly showed low counts of all three signatures. For AML, this is consistent with the finding published by the TCGA consortium that this cancer type has relatively stable genomes with an average of just one somatic copy number event per case [16].

**Table 1:** **Median signature scores for each cancer type**

**Cancer**	**Median NtAI**	**Median LST**	**Median HRD-LOH**	**Average ranking**	Receives first line platinum*
OV	24	20	15	1	Yes
LUSC	20	13	17	2	Yes
BLCA	16	11	9	3	Yes
HNSC	15	9	12	3	Yes
LUAD	15	10	11	3	Yes
BRCA	12	8	8	6	No
SKCM	9	6	7	7	Some
STAD	10	7	5	7	Yes
COAD	8	5	5	9	Yes
GBM	4	3	4	10	No
PRAD	4	6	2	11	No
KIRC	4	3	3	12	No
UCEC	3	2	2	13	No^$^
LAML	1	0	0	14	No
THCA	0	0	0	15	No^$^

*Based on treatment information available from Cancer Research UK, which can be found via http://www.cancerresearchuk.org/about-cancer/type/ ^$^Does not receive chemotherapy as first line, but may receive platinum-based chemotherapy for high stage or recurrent disease.

**Figure 3:**
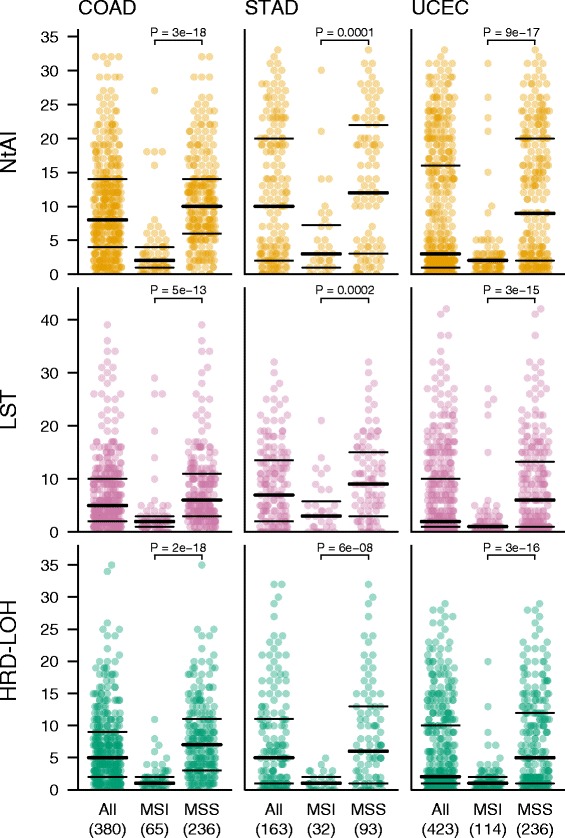
Distribution of NtAI, LST and HRD-LOH signature scores by MSI status in colon, gastric and endometrial cancer. Tumors are grouped by MSI (microsatellite instable) or MSS (microsatellite stable). Horizontal black lines indicate 25^th^, 50^th^ and 75^th^ percentiles.

We compared the median signature counts across cancer types and observed that the highest scoring cancer type for the NtAI and LST signatures was serous ovarian cancer (median NtAI = 24, LST = 20, Table 1) followed by squamous cell lung cancer. Both of these cancer types were high for HRD-LOH as well (LUSC = 17, OV = 15). Serous ovarian cancer is known to feature a high frequency of HR deficiency, often caused by homozygous loss of BRCA1 or BRCA2, either through germline or somatic inactivating mutations, or through promoter methylation, and there are defects in a large number of other DNA repair genes that could affect HR as well [17]. We then ranked the cancer types by average NtAI, LST and HRD-LOH scores (Table 1) and observed that the cancer types that are found at the top of the list typically include platinum-based chemotherapy as part of the first line standard of care, whereas the cancer types found with the lowest scores generally do not receive platinum as first line standard therapy. However, both advanced or recurrent endometrial and thyroid cancer may receive platinum-based therapy. With endometrial cancer, 50% shows NtAI of less than 3, and LST and HRD-LOH of less than 2. But 25% also shows NtAI scores ranging from 16 to 33, LST from 10 to 45, and HRD-LOH from 10 to 29. This suggests that while the majority are DNA repair proficient, there is a considerable subset of endometrial cancer patients that may have DNA repair deficiency, which may predispose to sensitivity to platinum-based therapy. When we investigated this further, we found that a considerable fraction of the endometrial cancers with low levels of genomic scars also tested positive for microsatellite instability (MSI). Indeed, almost all tumors with MSI showed low levels of genomic scars across colon, gastric and endometrial cancers, which were the three cancer types for which MSI was experimentally evaluated (Figure 3). This apparent tendency towards mutual exclusivity suggests that in MSI cancers, MSI arises before the genomic instability that generates genomic scars, and that there is no further selective advantage for the cancer cells to develop additional genomic instability.

Notably, breast cancer is in the middle of the ranked list. Breast cancer patients do not normally receive platinum-based chemotherapy in the adjuvant or first line metastatic setting. However, a subset of the triple-negative breast cancers has recently been shown be highly sensitive to cisplatin chemotherapy [7,10]. When we analyze the signature scores separately for the three subtypes, we do indeed find that TNBC shows much higher signature scores than either of the ER/PR+ and HER2+ subtypes (Additional file 1: Figure S1), with TNBC showing median NtAI, LST and HRD-LOH values of 27, 21.5 and 21, respectively. This is even higher than the values observed for serous ovarian cancer, suggesting a higher frequency of DNA repair deficiency.

### Comparing genomic scar signature scores

**Figure 4:**
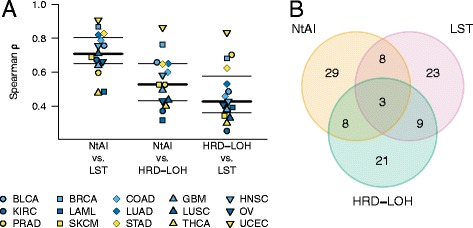
Comparison between signature scores. **A**: Spearman correlation coefficients for signature pairs. **B**: Venn diagram showing the overlap of genomic scars counted by the three signatures. All breakpoints measured by one or more signatures were counted, and each area gives the percentage of those breakpoints that were measured by each signature or combination of signatures.

To determine if tumors that scored high by one signature also scored high by the others, we first determined the Spearman correlation coefficient (ρ) between each pair of signature scores across all cancer types and within each cancer type (Figure 4A). We found that NtAI and LST generally showed a high correlation to each other (Spearman’s ρ = 0.87), with ρ > 0.7 in 8 of 15 cancer types. HRD-LOH shows a lower level of correlation with both NtAI (ρ = 0.81) and LST (ρ = 0.73), with ρ > 0.7 in just 2 of 15 cancer types for both NtAI and LST. Across cancer types, the three signatures showed the best correlation to each other in endometrial cancer, with ρ = 0.90 (NtAI vs. LST), 0.86 (NtAI vs. HRD-LOH), and 0.83 (LST vs. HRD-LOH). This could indicate that NtAI and LST measure overlapping genomic scars and therefore give high scores to more similar subpopulations of tumors, whereas the lower correlation to HRD-LOH suggests that it is counting genomic scars of a somewhat separate tumor subpopulation. To test this hypothesis, we compared the actual genomic aberrations measured by the three signatures. Here we found that on average only 3.1% of all measured copy number events were counted by all three signatures across cancer types (Figure 4B). Further, the number of chromosomal events counted by both NtAI and LST (11.4%) was no higher than the number counted by NtAI and HRD-LOH (11.4%) or LST and HRD-LOH (11.8%). This shows that the high correlation between NtAI and LST is not caused by an overlap in the individual events counted. Instead, the high correlation could indicate that the underlying repair defect generates multiple complex genomic aberration patterns, and that the different methods rely on capturing different non-overlapping aspects of these patterns to estimate the overall DNA repair competency.

### Association between genomic scar signatures and other measures of chromosomal instability

**Figure 5:**
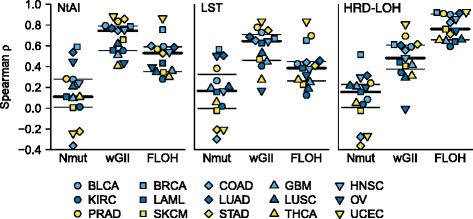
Comparison of signature scores to other measures of genome instability. Spearman correlation coefficients between each of the three HR signatures (NtAI, LST and HRD-LOH) and each of three alternative genomic signatures (wGII, FLOH and Nmut, see text for details).

As NtAI, LST and HRD-LOH can be considered a type of genomic instability score, we next compared the signature scores to three other previously published measures of chromosomal instability: the weighted genome integrity index (wGII) [18], the frequency of LOH (FLOH) [19], and the total number of mutations per sample (Nmut) [20], Figure 5. We observed that both NtAI and LST scores showed a good correlation with wGII (median ρ = 0.75 and 0.64, respectively), whereas HRD-LOH was lower, with a median ρ = 0.49. Correlation to FLOH was lower for both NtAI and LST (median ρ = 0.53 & ρ = 0.39), but HRD-LOH was considerably higher with median ρ = 0.76. This is not surprising, since HRD-LOH is measuring a type of LOH. The correlation to Nmut was much lower for all three signatures, with ρ = 0.11, 0.16 and 0.16 for NtAI, LST and HRD-LOH, respectively. This suggests that a simple measure of mutation counts is not a good surrogate for genomic scars as defined by either method, but that more general measures of chromosomal instability such as wGII and FLOH are correlated to these genomic scar-based approaches.

### Near-tetraploid tumors show increased NtAI and LST scores

Both the NtAI and LST signatures measure a genomic aberration pattern where a single allelic event may increase the score. As such, an increase in sample ploidy increases the amount of DNA that may experience genomic aberrations, and this may correlate with increased signature counts. Indeed, in the original publication of LST [13], Popova and colleagues showed that the threshold for calling HR deficiency using LST should be set higher for near-tetraploid tumors compared to near-diploid tumors, at 20 versus 15. The HRD-LOH score specifically measures LOH, the complete loss of one parental allele. As ploidy increases beyond diploid, complete LOH in the absence of selective pressure becomes less likely in a purely probabilistic sense, as the generation of a region of LOH requires multiple events of genomic loss at the same site. Thus, the generation of new HRD-LOH events is theoretically less likely to occur as ploidy increases.

When we compared the signature scores in near-diploid versus near-tetraploid cancers (see Methods), we do indeed find that for both NtAI and LST, the mean signature score is significantly increased in near-tetraploid samples for 11 and 12 of 15 cancer types, respectively (Additional file 2: Figure S2). This was true for only 6 of 15 cancer types for HRD-LOH, and additionally we found a significant decrease in the HRD score in near-tetraploid ovarian cancer and stomach adenocarcinoma (Additional file 3: Figure S3). The fact that some cancer types showed significantly increased HRD-LOH scores in near-tetraploid tumors could indicate that these cancer types are prone to undergo a late tetraploidization step in an already established cancer, where parental alleles have already been lost due to chromosomal instability in a diploid state.

### Tumors with p53 mutations show increased NtAI, LST and HRD-LOH scores

As both TNBC and serous ovarian cancer commonly have p53 mutations, we tested if p53 status itself would be associated with increased scores of NtAI, LST and HRD-LOH. We examined cancer types with at least 20 samples with P53 loss, and at least 20 samples with wtP53. We found that all showed a significant increase in the scores of all signatures in mP53, with the exception of melanoma and stomach adenocarcinoma for LST, and melanoma for HRD-LOH (Additional file 4: Figure S4). As p53 loss is often associated with genomic instability and increased ploidy states, we also compared the p53 status of near-diploid and near-tetraploid samples. Overall, we found that 44% of near-diploid tumors showed p53 mutations (1177 out of 2690), whereas 74% of near-tetraploid tumors showed p53 mutation (1443 out of 1953. Odds ratio = 3.64, p < 0.0001, Fisher’s exact test).

### Stage and grade are not consistently associated with high NtAI, LST and HRD-LOH scores

Stage was available for 12 of 15 cancer types. When we compared NtAI, LST and HRD-LOH in samples with low (stage 1 + 2) versus high stage (stage 3 + 4), we found that high stage was significantly associated with higher NtAI and LST scores in breast and head and neck cancer, and with higher of all three scores in colon, renal, and endometrial cancer (Additional file 5: Figure S5). Grade was available for only 6 of 15 cancer types. Of these, all three scores were significantly higher in high grade (3–4) bladder, renal and endometrial cancer. However, for stomach adenocarcinoma all three scores showed a significantly lower signature score in high grade tumors (Additional file 6: Figure S6).

### Smoking associates with high NtAI, LST and HRD-LOH scores in lung adenocarcinoma and head and neck cancer

Tobacco smoke is a known carcinogen, and highly associated with lung cancer and head and neck cancer. If tobacco smoke also causes increased levels of DNA damage that results in genomic scaring measured by either predictor, we would expect to find higher counts of NtAI, LST, and HRD-LOH in patients that smoke compared to non-smokers. Clinical annotations regarding the patient smoking history were available for adeno and squamous cell lung cancer, head and neck cancer, and bladder cancer. We grouped patients currently smoking with patients that stopped smoking within the past 15 years (“smokers”), and compared these to patients that never smoked, or stopped smoking at least 15 years ago (“non-smokers”). This showed a clear increase in the median NtAI, LST and HRD-LOH counts for smokers relative to non-smokers in head and neck cancer patients (median NtAI: 17 versus 12; LST: 10 versus 8, HRD-LOH: 13 versus 9), and in lung adenocarcinoma cancer (median NtAI: 18 versus 12, LST: 13 versus 8, HRD-LOH: 14 versus 9). No significant difference was found for bladder or squamous cell lung cancer (Additional file 7: Figure S7).

## Discussion

In this study we performed a comprehensive analysis of 5371 tumors representing 15 cancer types, to investigate the distribution of previously characterized genomic scars associated with HR deficiency, a DNA repair deficiency that causes increased sensitivity to platinum-based genotoxic chemotherapy and PARP inhibitors [15]. We showed that the three signatures of genomic scars demonstrate only limited overlap in the chromosomal events identified by each signature, yet there is a good correlation between the total number of events they identify in each sample. This suggests that the genomic scars they measure are caused by identical or related DNA repair deficiencies, likely loss of different elements in the HR pathway. Unfortunately it is not possible based on the current work to establish the true biological basis of these genomic scars. However, if the difference between signature scores indeed represents deficiencies in different elements of the HR pathway, then it is plausible that sensitivity to therapy will be particularly high when all signatures are high. Conversely, if the signatures all represent the same underlying biology, then detection error will determine sensitivity, and one high scoring signature could potentially predict for response as well as if all three signatures are high. Further studies are required to fully elucidate the true biological nature of these signatures. Nevertheless, all three signatures identify considerable subpopulations of tumors with high signature scores within most cancer types, including those not regularly treated with platinum chemotherapy. This might indicate that small but identifiable subpopulations of cancer patients exists that could potentially benefit from genotoxic therapy, but are currently not receiving it as standard of care.

Although it has been shown in breast and ovarian cancer that increased numbers of the genomic scars counted by all three signatures is associated with response to platinum-based therapies [8,13,14], this has not been shown for any other cancer type. It is plausible that in other cancer types, genomic scars counted by either method may be caused by completely different mechanisms, and hence tumors with high scores may or may not show increased sensitivity to platinum-based therapies or PARP inhibitors. Nevertheless, the observation of high median scores in tumor types for which platinum is currently used as standard of care suggests that at least in some additional tumor types, these signatures may indeed predict for platinum response. This can only be fully resolved through future prospective trials.

Across cancer types, the tumors with high signature scores often harbor p53 inactivation and a near-tetraploid genome. Loss of p53 is known to cause genome instability in part through deficiency in the G1/S checkpoint [21] and render cells permissive of tetraploidization [22]. However, it has previously been reported that p53 loss is associated with platinum resistance in ovarian cancer [23], and that in basal-like breast cancer, tetraploidization was more prevalent in tumors with wildtype BRCA1 than in tumors harboring inactivation of BRCA1 [13]. This suggests that the genomic scars caused by p53 inactivation and tetraploidization, and which are measured by NtAI, LST or HRD-LOH, arise through mechanisms that are unrelated to the DNA repair deficiencies that are associated with sensitivity to genotoxic agents. Further studies should therefore evaluate whether the signature thresholds used to predict therapy response should be set higher for tumors with p53 inactivation and/or tetraploid genomes.

We observe higher values of NtAI, LST and HRD-LOH for four cancer types often associated with tobacco smoking: bladder, lung adenocarcinoma and squamous cell cancer, and head and neck cancer. This suggests that continuous exposure to a carcinogen that causes increased levels of DNA damage may result in a higher background level of copy number aberrations. However, when we compared the levels of genomic scars in current or recent smokers to those in non-smokers, we observed lower levels of NtAI, LST and HRD-LOH in the non-smoking population in lung adenocarcinoma cancer and head and neck cancer, whereas there was no difference between these groups in squamous lung and bladder cancer. It is not clear why such a difference is found, but it is possible that in lung adenocarcinoma and head and neck cancer, smoking induces a cancer type that is different from cancers arising outside of a smoking context, whereas the mature phenotype of squamous lung and bladder cancer may be more uniform regardless of exposure to carcinogens from smoking.

In our investigation of the distribution of genomic scars across cancer types, endometrial cancer was of particular interest. In endometrial cancer, the overall level of genomic scars was very low, with a median of 3/2/2 for NtAI/LST/HRD-LOH scores. However, a significant subset of microsatellite stable tumors showed a strong increase in the genomic scar levels. In addition, there was a highly significant association between higher levels of genomic scars and both p53 mutation (Additional file 4: Figure S4) and higher grade (Additional file 6: Figure S6). As platinum chemotherapy is widely used as standard of care for advanced endometrial carcinoma, these results may warrant further exploration as to whether the inclusion of a genomic scar-based biomarker might improve upon the stratification of these patients for adjuvant chemotherapy.

## Conclusions

Personalized medicine in cancer care holds great promise, but unfortunately most drugs currently in clinical practice are not paired with specific biomarkers, and it is therefore not possible to predict their effect on a given patient prior to therapy. Platinum agents and other genotoxic agents work by inducing intolerable levels of DNA damage into cancer cells. These agents have proven highly successful and remain the mainstay of cancer chemotherapy across many cancer types, but to this date the identification of biomarkers for efficacy of genotoxic drugs has proven elusive. As cancer cells sensitive to treatment with genotoxic agents have reduced capacity to tolerate DNA damage, possibly through loss of DNA repair factors, identifying biomarkers that indicate cancer-specific DNA repair capacity may prove a useful proxy for predicting sensitivity to genotoxic therapy. Here, the concept of a DNA scar becomes potentially useful as a measure of DNA repair capacity. If the use of the genomic signatures of DNA scarring can be validated in prospective clinical trials, this may turn already widely used and highly potent drugs into effective targeted agents available to any cancer patient with a sensitive tumor, regardless of its site of origin.

## Methods

### Datasets

From the TCGA data portal, dbGap accession no. phs000178.v5.p5, we obtained Affymetrix SNP6 genotyping data and clinical information for 5502 unique cancer samples representing 15 distinct cancer types, listed in Table 2.

**Table 2:** **Number of samples per cancer type in the present analysis**

**TCGA code**	**Cancer type**	**Number of samples**
BLCA	Bladder urothelial carcinoma	127
BRCA	Breast invasive carcinoma	877
COAD	Colon adenocarcinoma	380
GBM	Glioblastoma multiforme	456
HNSC	Head and neck squamous cell carcinoma	294
KIRC	Kidney renal clear cell carcinoma	433
LAML	Acute myeloid leukemia	153
LUAD	Lung adenocarcinoma	305
LUSC	Lung squamous cell carcinoma	241
OV	Ovarian serous cystadenocarcinoma	512
PRAD	Prostate adenocarcinoma	325
SKCM	Skin cutaneous melanoma	244
STAD	Stomach adenocarcinoma	163
THCA	Thyroid carcinoma	438
UCEC	Uterine corpus endometrial carcinoma	423

### Data processing

All data analysis was performed in the R statistical environment, version 3.0.1. Affymetrix SNP6 data from paired tumor-normal samples were normalized and preprocessed using the Aroma Affymetrix CRMAv2 algorithm [24], and the B-allele fraction (BAF) was adjusted using the CalMaTe and TumorBoost algorithms [25,26]. Tumor copy number aberrations, ploidy and normal cell contamination was determined using ASCAT [27]. Tumors with aberrant cell fraction (ACF) below 0.36 was excluded from the analysis.

### Implementing genomic signatures

We implemented algorithms to quantify each of the genomic scar signatures in R. All signature scores are available in Additional file 8: Table S1, and the code is available in Additional file 9. The signatures were implemented following the directions described in the original publications [8,13,14] with two exceptions: 1) The original publication describing HRD-LOH [14] excluded chromosome 17 because LOH on chromosome 17 in the ovarian cancer samples is ubiquitous and for this reason did not provide independent information. We decided to not exclude chromosome 17, as chromosome 17 is not ubiquitously lost in all cancer types, and therefore may provide independent information in some tumor samples. 2) In the original publication describing NtAI [8], we counted all allelic imbalance events that extended to the telomere, if they did not span the centromere. In subsequent work, we have found that this occasionally biases samples with an uneven chromosome count. If e.g. triploid chromosomes have an independent interstitial copy number event of sufficient size between telomere and centromere, it was counted as an NtAI event. This results in an overrepresentation of tumors with an uneven copy number among high NtAI cases, which has been corrected in the method used for the present study. In the implementation of the NtAI method used here, we determine the major copy number state for each chromosome independently, defined as the copy number state of the majority of the chromosome that is greater than zero. We then count NtAI events only if these deviate from the main copy number state of a given chromosome, while taking allelic contribution into account (Additional file 1: Figure S1). Mutation data to determine the total number of mutations (Nmut) was defined as the number of called base substitutions, indels, and dinucleotide mutations [20], and was based on the curated dataset provided in the supplementary data from [28]. The frequency of LOH (FLOH) was determined as described [19].

### Sample ploidy estimate

The ploidy of each tumor is determined as part of the ASCAT modeling of the log_2_ copy number data and the B-allele frequency data. DNA index is defined as the tumor ploidy divided by the expected ploidy of 2. Based on the hypothesis that duplication of the whole genome during cancer progression is the most common initiation event for aneuploidy [29], samples with a DNA index > 1.2 are considered near-tetraploid in a manner similar to [13], whereas tumors with DNA index < 1.2 are considered as near-diploid.

### Data analysis

We used Spearman’s rank correlation coefficient (ρ) to measure concordance between continuous signature scores. The Wilcoxon rank sum test was used to test for increase in signature scores by ploidy. All p-values are two-sided.

## Endnote

^a^In the original publication [14] this method was referred to as “HRD”. This term is currently used to describe a score that combines NtAI, LST and HRD [30], and subsequently the recognised term for the original method described in [14] is currently “HRD-LOH”, which is therefore also used throughout this text.

## Additional files

Additional file 1: Figure S1. The effect of sample ploidy on NtAI calling. A: Scatter plot showing updated versus original NtAI score per sample, based on the TCGA ovarian cancer cohort. The colors and shapes indicate the ploidy state of the tumor. Tumors with ploidy state below 2 or above 4 are shown as unfilled black circles. Additional file 2: Figure S2. Signature scores in breast cancer subtypes Distribution of signature scores in ER-positive, HER2-positive and triple negative breast cancers. Additional file 3: Figure S3. Signature scores by ploidy. Mean signatures scores for near-diploid and near-tetraploid tumors across cancer types. A: NtAI, B: LST, C: HRD-LOH. Sample ploidy is defined as DNA index < 1.2 for near-diploid, or > 1.2 for near-tetraploid. Additional file 4: Figure S4. Signature scores by p53. Distribution of signature scores in tumors with wildtype and mutant p53. Additional file 5: Figure S5. Signature scores by stage. Distribution of signature scores by tumor stage. Low stage is defined as stage 1–2; high stage is defined as stage 3–4. Additional file 6: Figure S6. Signature scores by grade. Distribution of signature scores by tumor grade. Low grade is defined as grade 1–2; high grade is defined as grade 3–4. Additional file 7: Figure S7. Signature scores by smoking status. Distribution of signature scores by smoking status. A non-smoker is defined as a never-smoker or non-smoker for at least 15 years. A smoker is defined as a current or recent smoker (<15 years). Additional file 8: Table S1. The calculated signature scores along with other measures of chromosomal instability are provided for all tumors included in the present analysis. Additional file 9:
**Supplementary methods.** R source code with implementations of the algorithms to quantify NtAI, LST and HRD-LOH.

## Abbreviations

FLOH: Frequency of loss of heterozygosity
HR: Homologous recombination
HRD: Homologous recombination deficiency
LOH: Loss of heterozygosity
LST: Large scale transitions
MSI: Microsatellite instability
NtAI: Number of telomeric allelic imbalance
TCGA: The cancer genome atlas
TNBC: Triple negative breast cancer

## References

[1] Ciriello G, Miller ML, Aksoy BA, Senbabaoglu Y, Schultz N, Sander C (2013). Emerging landscape of oncogenic signatures across human cancers. Nature Genetics.

[2] Lengauer C, Kinzler KW, Vogelstein B (1998). Genetic instabilities in human cancers. Nature.

[3] Curtin NJ (2012). DNA repair dysregulation from cancer driver to therapeutic target. Nat Rev Cancer.

[4] Tassone P, Tagliaferri P, Perricelli A, Blotta S, Quaresima B, Martelli ML (2003). BRCA1 expression modulates chemosensitivity of BRCA1-defective HCC1937 human breast cancer cells. Br J Cancer.

[5] Farmer H, McCabe N, Lord CJ, Tutt ANJ, Johnson DA, Richardson TB (2005). Targeting the DNA repair defect in BRCA mutant cells as a therapeutic strategy. Nature.

[6] Bryant HE, Schultz N, Thomas HD, Parker KM, Flower D, Lopez E (2005). Specific killing of BRCA2-deficient tumours with inhibitors of poly(ADP-ribose) polymerase. Nature.

[7] Silver DP, Richardson AL, Eklund AC, Wang ZC, Szallasi Z, Li Q (2010). Efficacy of neoadjuvant cisplatin in triple-negative breast cancer. J Clin Oncol.

[8] Birkbak NJ, Wang ZC, Kim JY, Eklund AC, Li Q, Tian R (2012). Telomeric allelic imbalance indicates defective DNA repair and sensitivity to DNA-damaging agents. Cancer Discovery.

[9] Yang D, Khan S, Sun Y, Hess K, Shmulevich I, Sood AK (2011). Association of BRCA1 and BRCA2 mutations with survival, chemotherapy sensitivity, and gene mutator phenotype in patients with ovarian cancer. JAMA.

[10] Byrski T, Dent R, Blecharz P, Foszczynska-Kloda M, Gronwald J, Huzarski T (2012). Results of a phase II open-label, non randomized trial of cisplatin chemotherapy in patients with BRCA1-positive metastatic breast cancer. Breast Cancer Res.

[11] Iglehart JD, Silver DP (2009). Synthetic lethality–a new direction in cancer-drug development. N Engl J Med.

[12] Tutt A, Robson M, Garber JE, Domchek SM, Audeh MW, Weitzel JN (2010). Oral poly(ADP-ribose) polymerase inhibitor olaparib in patients with BRCA1 or BRCA2 mutations and advanced breast cancer: a proof-of-concept trial. Lancet.

[13] Popova T, Manie E, Rieunier G, Caux-Moncoutier V, Tirapo C, Dubois T (2012). Ploidy and large-scale genomic instability consistently identify basal-like breast carcinomas with BRCA1/2 inactivation. Cancer Res.

[14] Abkevich V, Timms KM, Hennessy BT, Potter J, Carey MS, Meyer LA (2012). Patterns of genomic loss of heterozygosity predict homologous recombination repair defects in epithelial ovarian cancer. Br J Cancer.

[15] Watkins JA, Irshad S, Grigoriadis A, Tutt AN (2014). Genomic scars as biomarkers of homologous recombination deficiency and drug response in breast and ovarian cancers. Breast Cancer Res.

[16] The Cancer Genome Atlas Research Network (2013). Genomic and epigenomic landscapes of adult De novo acute myeloid leukemia. N Engl J Med.

[17] Cancer Genome Atlas Research Network (2011). Integrated genomic analyses of ovarian carcinoma. Nature.

[18] Burrell RA, McClelland SE, Endesfelder D, Groth P, Weller M-C, Shaikh N (2013). Replication stress links structural and numerical cancer chromosomal instability. Nature.

[19] Wang ZC, Birkbak NJ, Culhane AC, Drapkin R, Fatima A, Tian R (2012). Profiles of genomic instability in high-grade serous ovarian cancer predict treatment outcome. Clin Cancer Res.

[20] Birkbak NJ, Kochupurakkal B, Izarzugaza JMG, Eklund AC, Li Y, Liu J (2013). Tumor mutation burden forecasts outcome in ovarian cancer with BRCA1 or BRCA2 mutations. PLoS One.

[21] Halazonetis TD, Gorgoulis VG, Bartek J (2008). An oncogene-induced DNA damage model for cancer development. Science.

[22] Fujiwara T, Bandi M, Nitta M, Ivanova EV, Bronson RT, Pellman D (2005). Cytokinesis failure generating tetraploids promotes tumorigenesis in p53-null cells. Nat Cell Biol.

[23] Martin LP, Hamilton TC, Schilder RJ (2008). Platinum resistance: the role of DNA repair pathways. Clin Cancer Res.

[24] Bengtsson H, Wirapati P, Speed TP (2009). A single-array preprocessing method for estimating full-resolution raw copy numbers from all Affymetrix genotyping arrays including GenomeWideSNP 5 & 6. Bioinformatics.

[25] Ortiz-Estevez M, Aramburu A, Bengtsson H, Neuvial P, Rubio A: CMT (2012). A method and software to improve allele-specific copy number of SNP arrays for downstream segmentation. Bioinformatics.

[26] Bengtsson H, Neuvial P, Speed TP (2010). TumorBoost: Normalization of allele-specific tumor copy numbers from a single pair of tumor-normal genotyping microarrays. BMC Bioinformatics.

[27] Van Loo P, Nordgard SH, Lingjærde OC, Russnes HG, Rye IH, Sun W (2010). Allele-specific copy number analysis of tumors. Proc Natl Acad Sci U S A.

[28] Alexandrov LB, Nik-Zainal S, Wedge DC, Aparicio SAJR, Behjati S, Biankin AV (2013). Signatures of mutational processes in human cancer. Nature.

[29] Storchova Z, Kuffer C (2008). The consequences of tetraploidy and aneuploidy. J Cell Sci.

[30] Timms KM, Abkevich V, Hughes E, Neff C, Reid J, Morris B (2014). Association of BRCA1/2 defects with genomic scores predictive of DNA damage repair deficiency among breast cancer subtypes. Breast Cancer Res..

