# Supplementary material for: Pan-cancer analysis of genomic scar signatures associated with homologous recombination deficiency suggests novel indications for existing cancer drugs
Source: Biomark Res. 2015 May 1;3:9. doi: 10.1186/s40364-015-0033-4 (PMC4443545; doi:10.1186/s40364-015-0033-4)
Supplement: Additional file 1: Figure S1. — The effect of sample ploidy on NtAI calling. A: Scatter plot showing updated versus original NtAI score per sample, based on the TCGA ovarian cancer cohort. The colors and shapes indicate the ploidy state of the tumor. Tumors with ploidy state below 2 or above 4 are shown as unfilled black circles. [file 40364_2015_33_MOESM1_ESM.pdf]

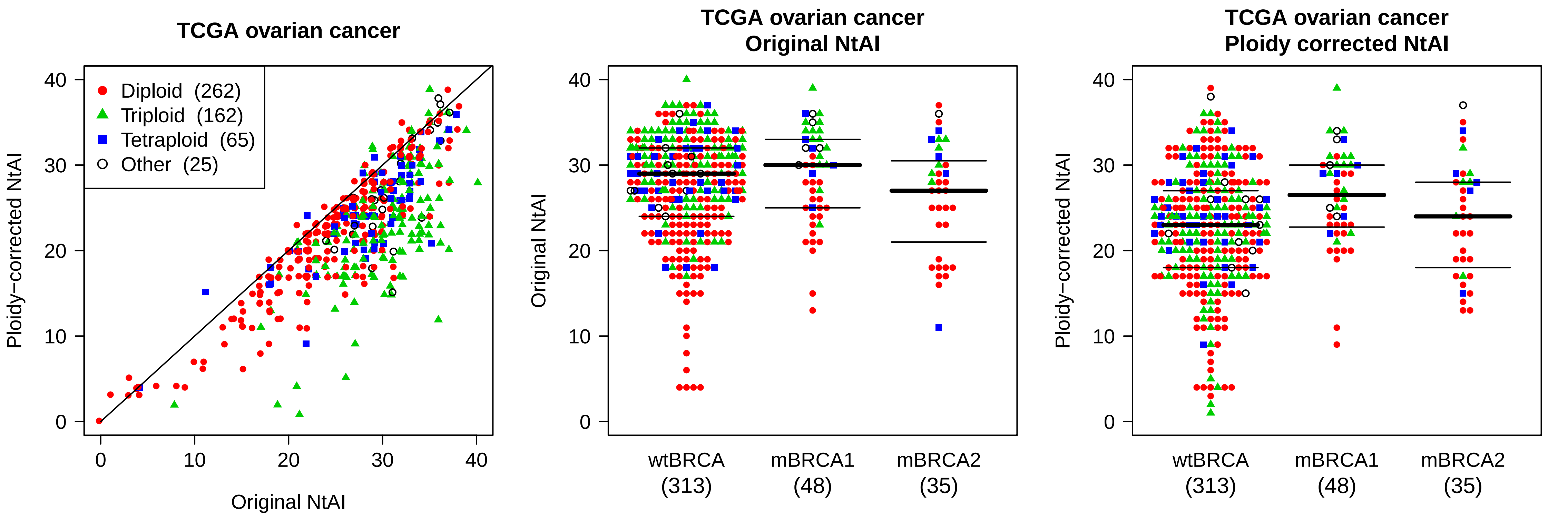
TCGA ovarian cancer
Diploid (262)
Triploid (162)
Tetraploid (65)
Other (25)
Ploidy−corrected NtAI
Original NtAI
TCGA ovarian cancer
Original NtAI
Original NtAI
wtBRCA (313)
mBRCA1 (48)
mBRCA2 (35)
TCGA ovarian cancer
Ploidy corrected NtAI
Ploidy−corrected NtAI
wtBRCA (313)
mBRCA1 (48)
mBRCA2 (35)
