# Supplementary figures and images for: Pan-cancer analysis of genomic scar signatures associated with homologous recombination deficiency suggests novel indications for existing cancer drugs
Source: Biomark Res. 2015 May 1;3:9. doi: 10.1186/s40364-015-0033-4 (PMC4443545; doi:10.1186/s40364-015-0033-4)

# BRCA

NtAI

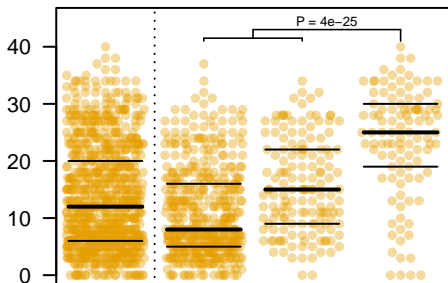

LST

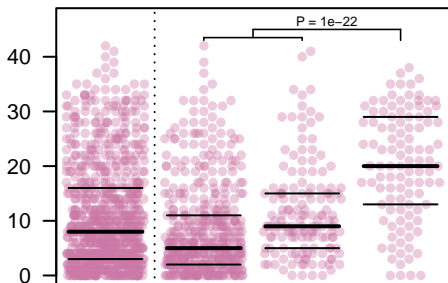

HRD-LOH

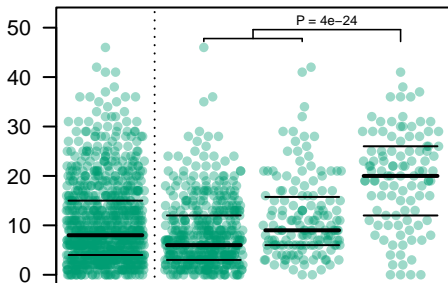

All  
(877)

ER  
(481)

HER2  
(150)

TNBC  
(105)

Supplement: Additional file 2: Figure S2. — Signature scores in breast cancer subtypes Distribution of signature scores in ER-positive, HER2-positive and triple negative breast cancers. [file 40364_2015_33_MOESM2_ESM.pdf]

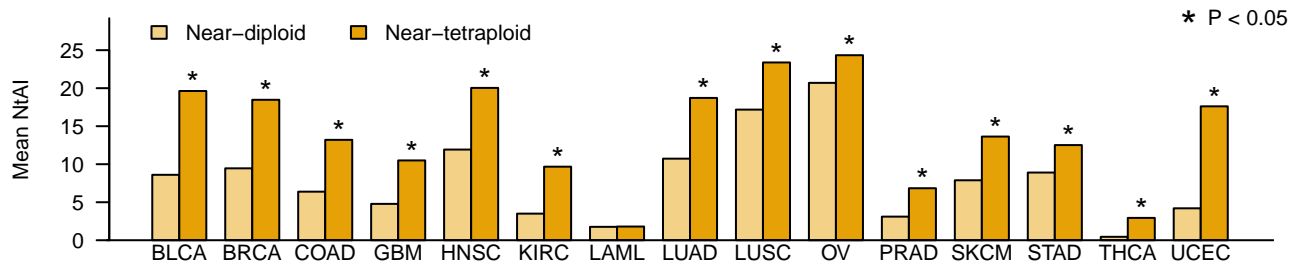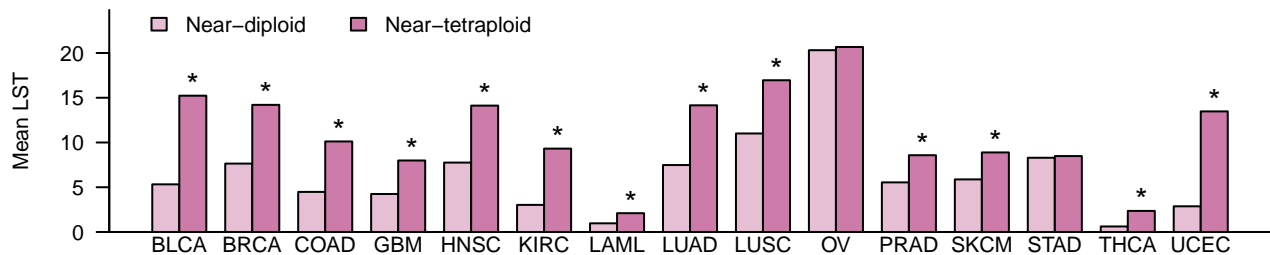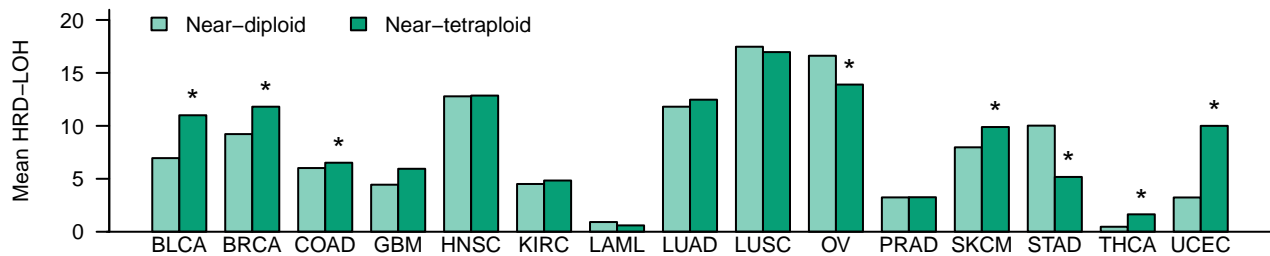

Supplement: Additional file 3: Figure S3. — Signature scores by ploidy. Mean signatures scores for near-diploid and near-tetraploid tumors across cancer types. A: NtAI, B: LST, C: HRD-LOH. Sample ploidy is defined as DNA index < 1.2 for near-diploid, or > 1.2 for near-tetraploid. [file 40364_2015_33_MOESM3_ESM.pdf]

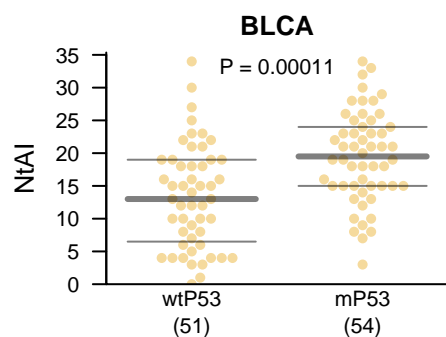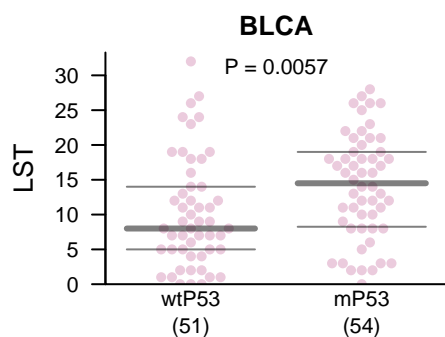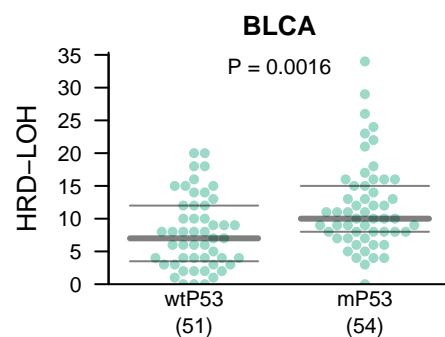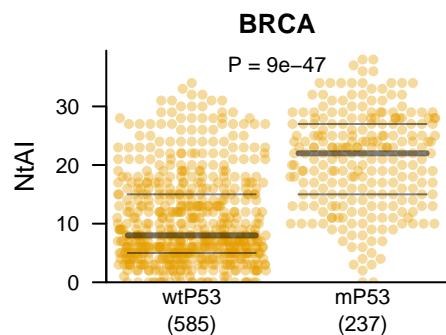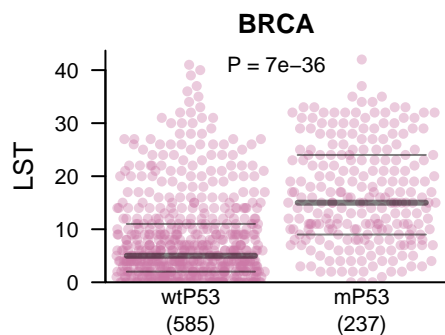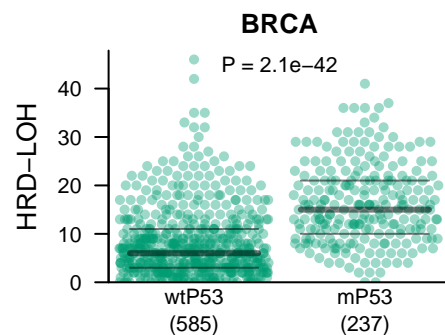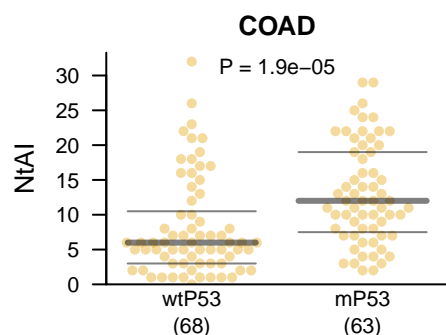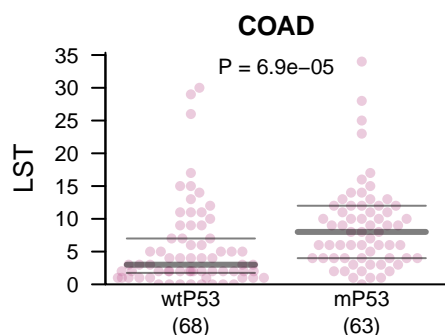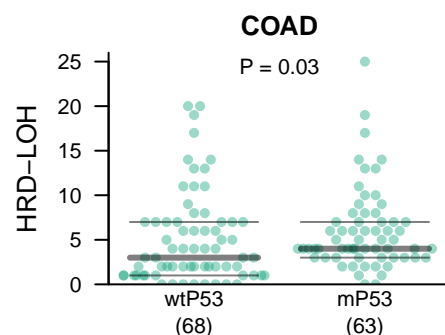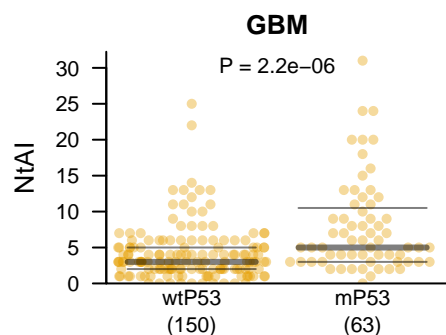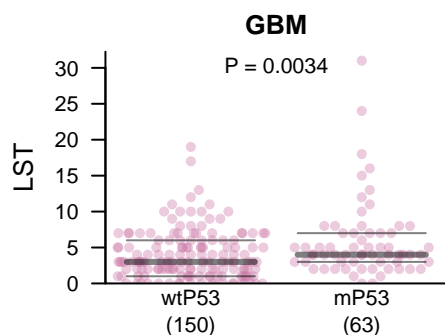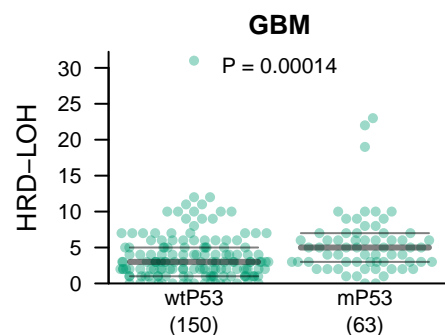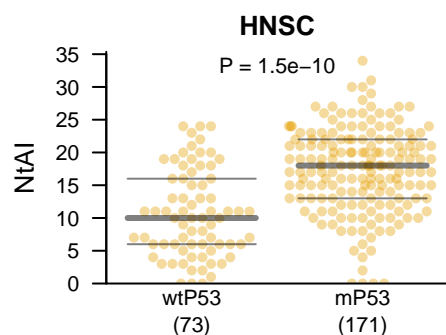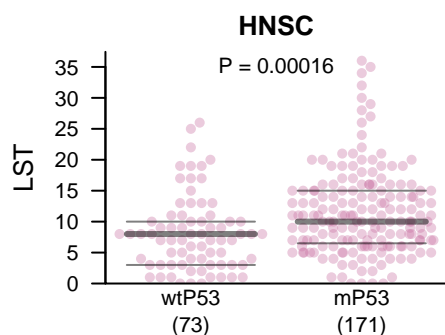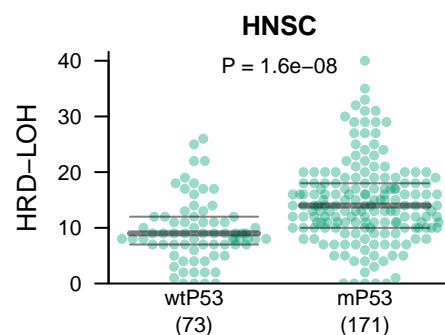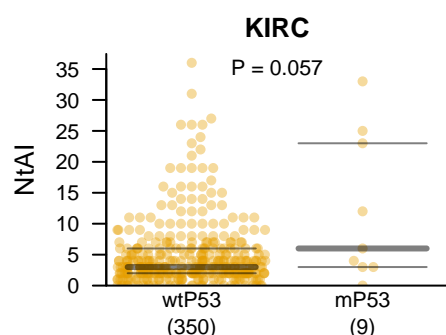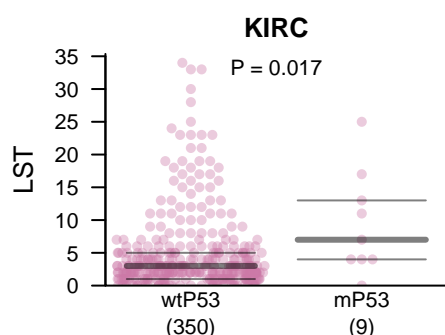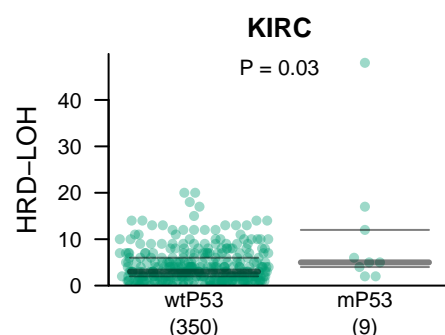

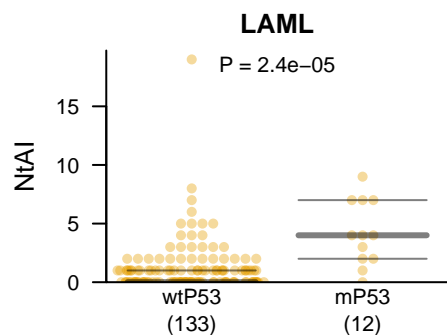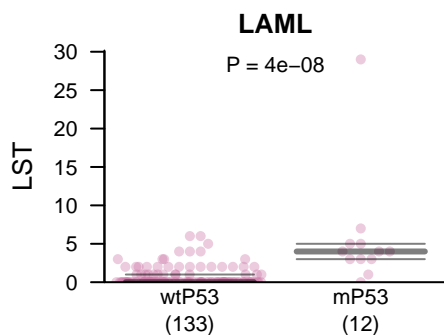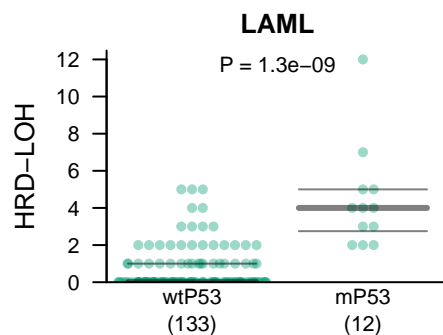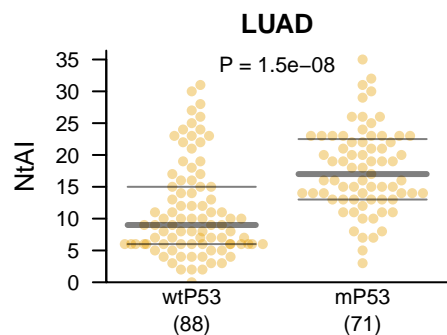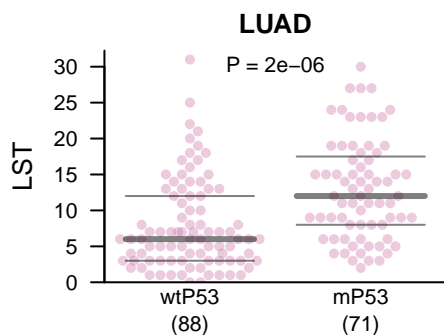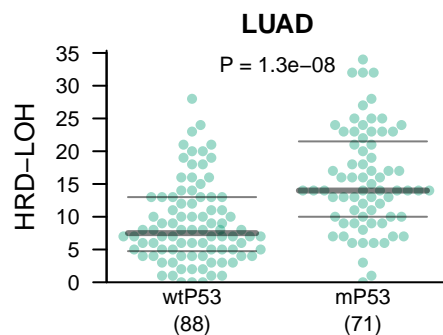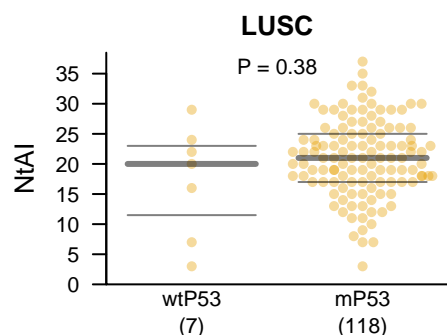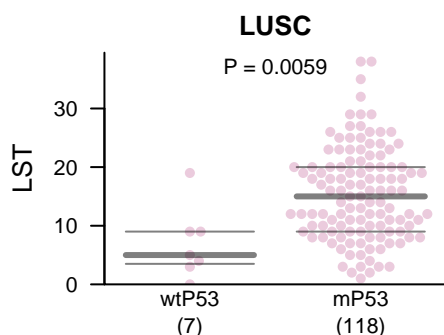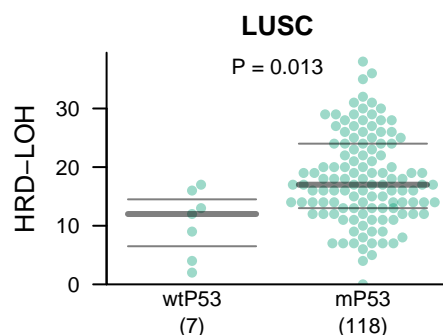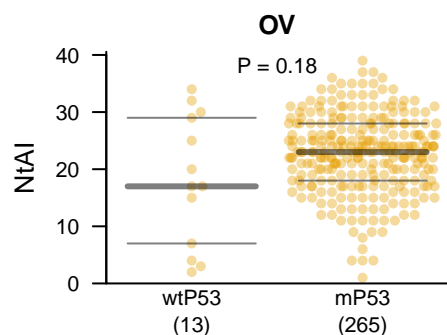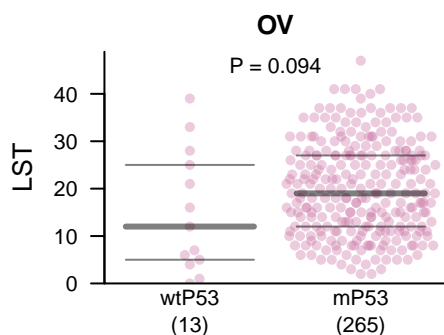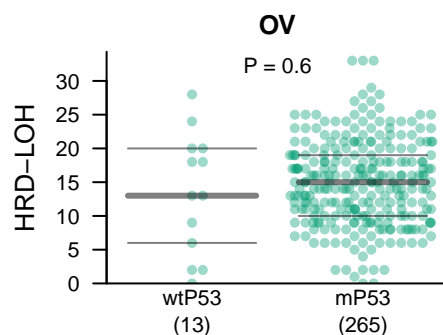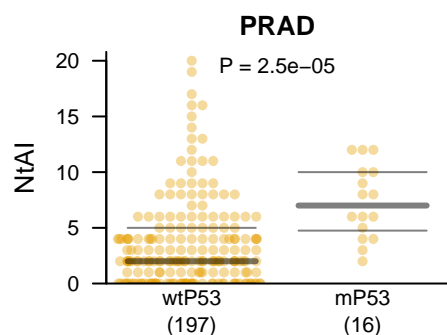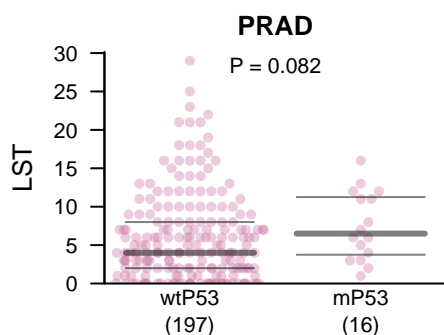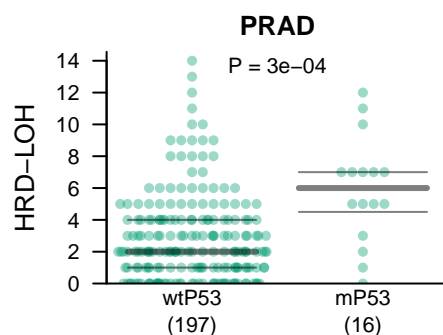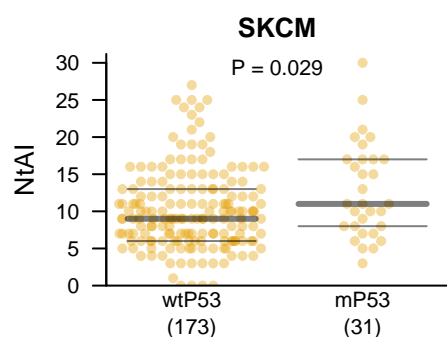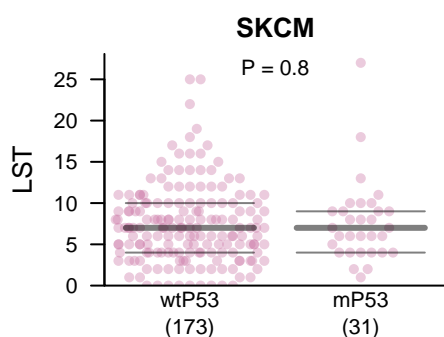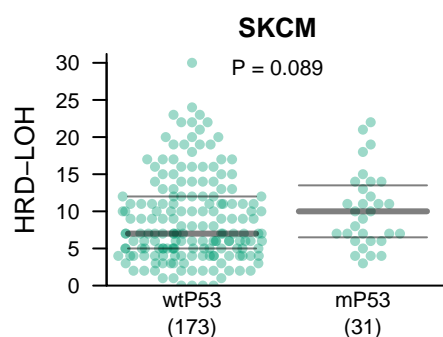

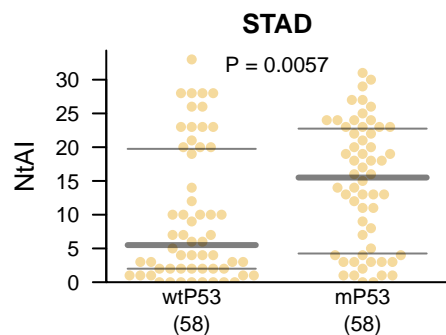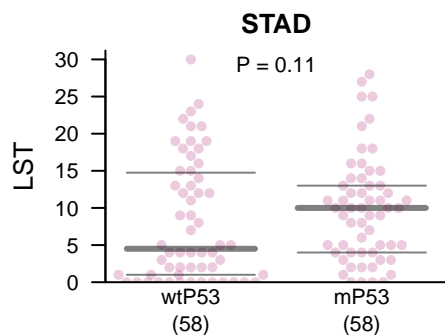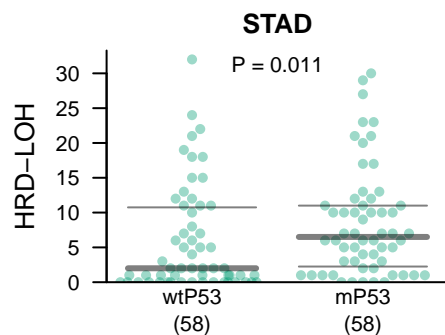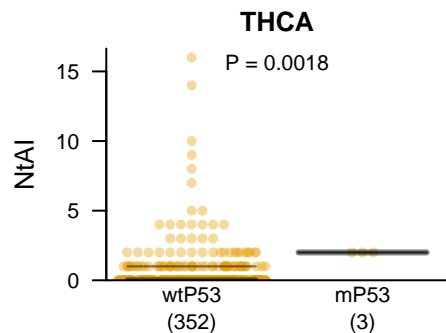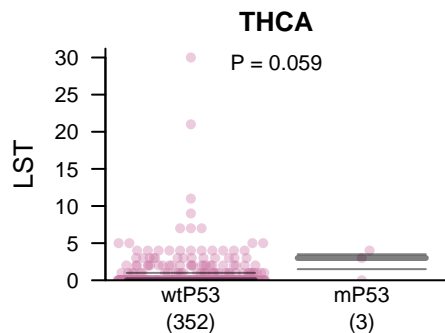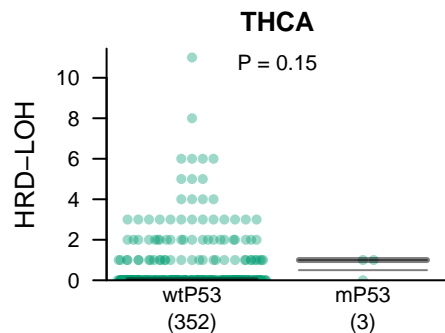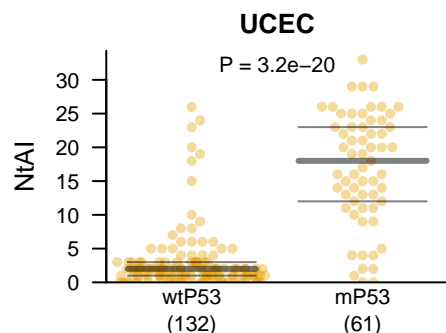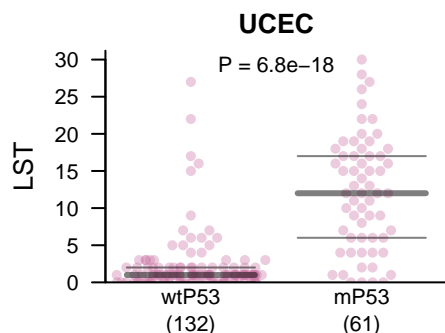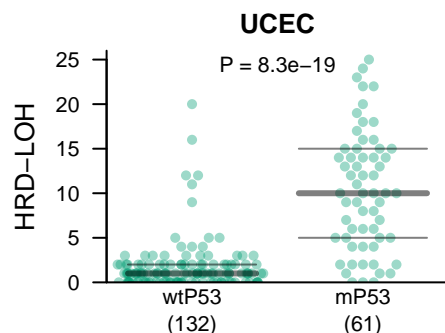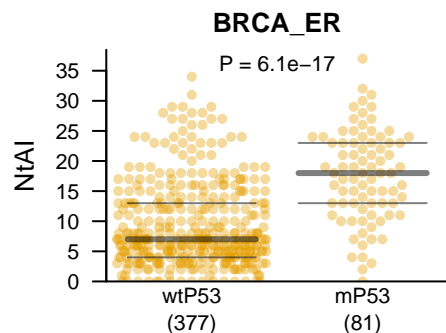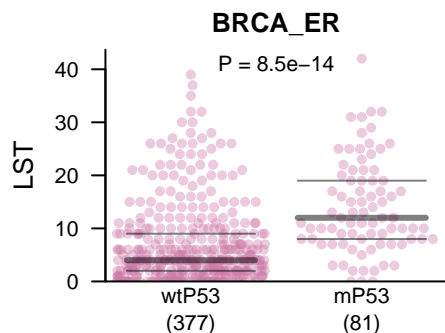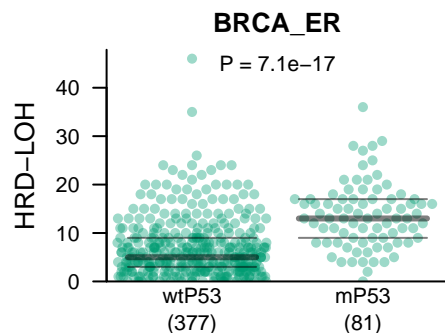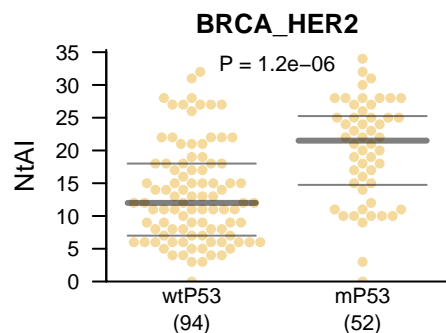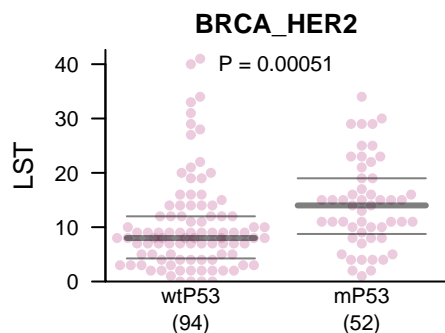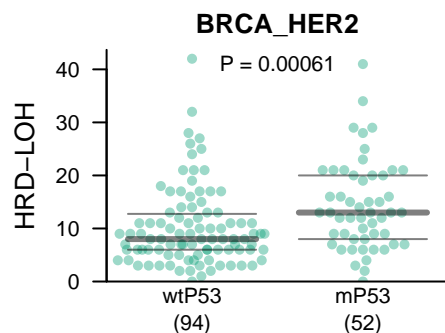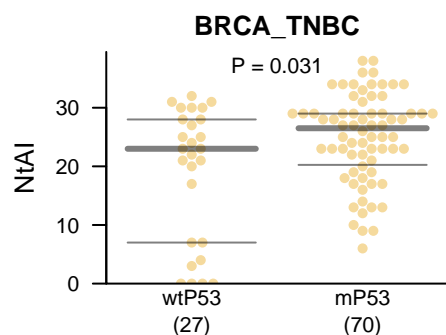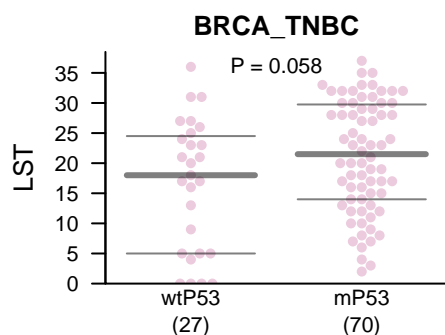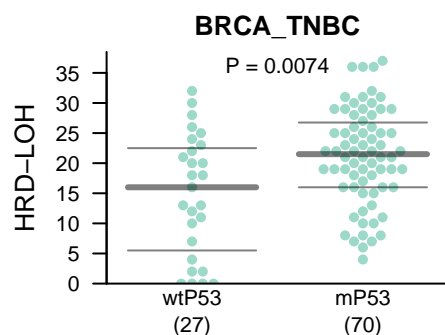

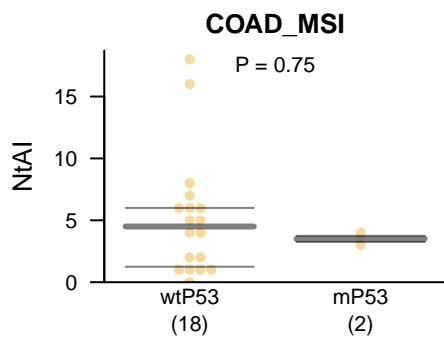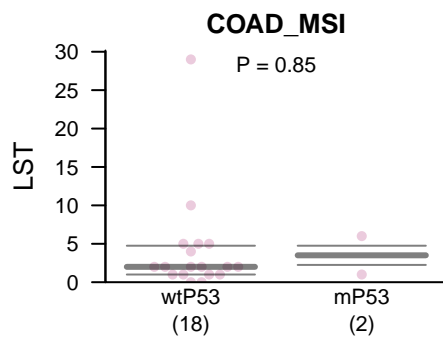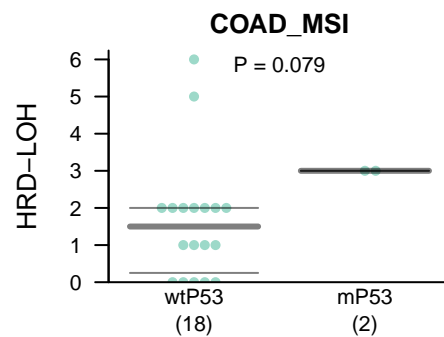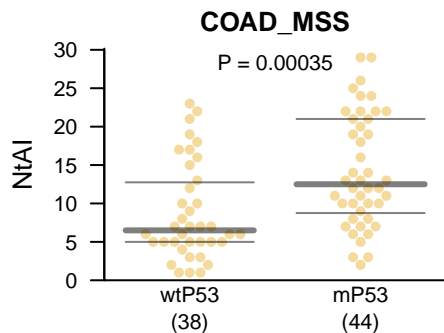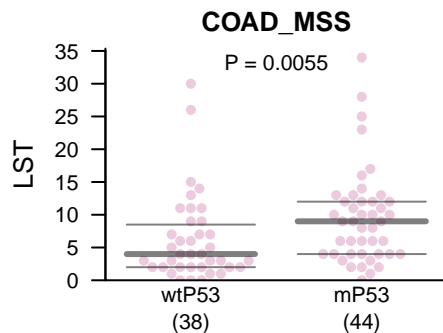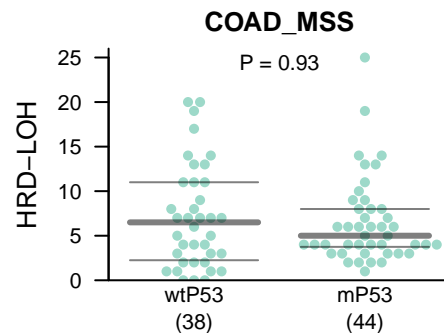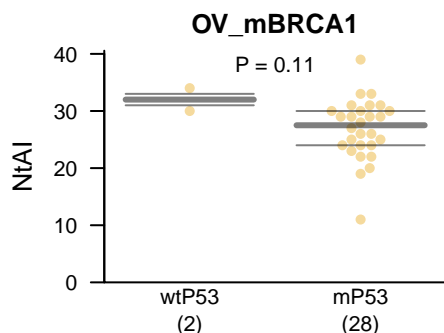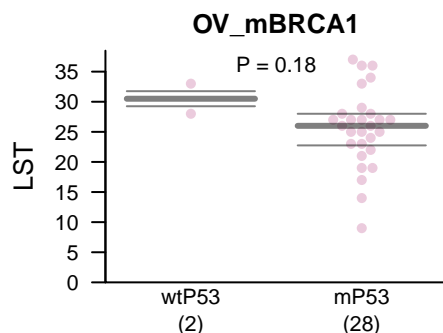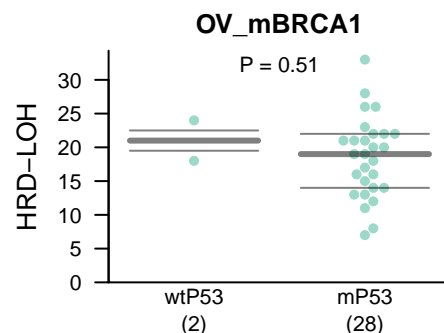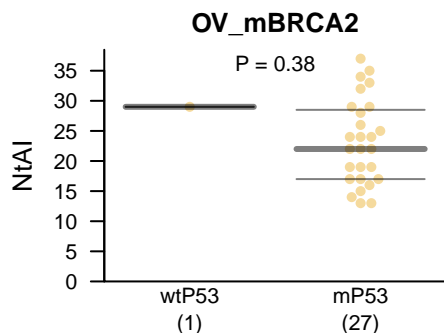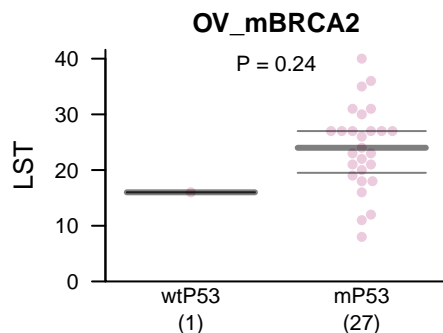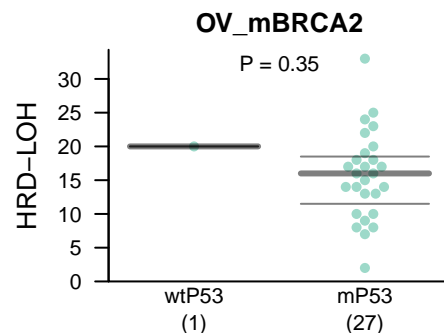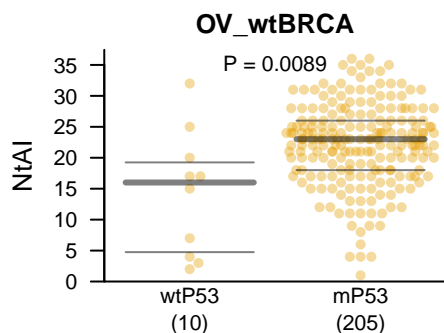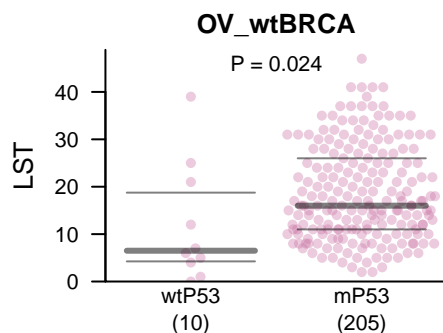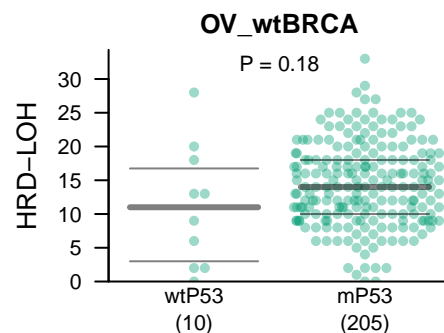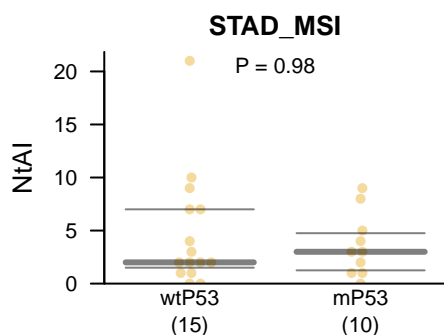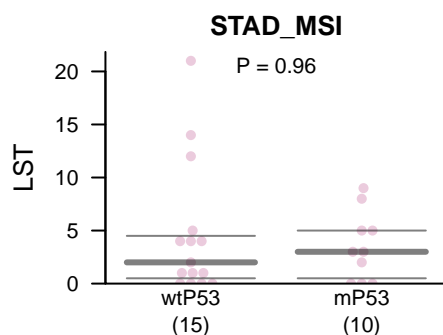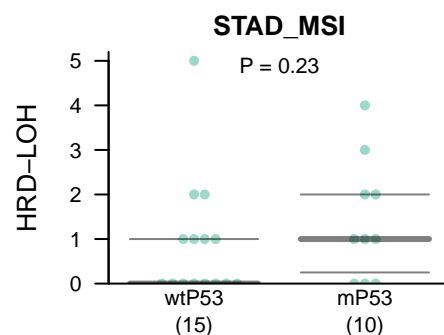

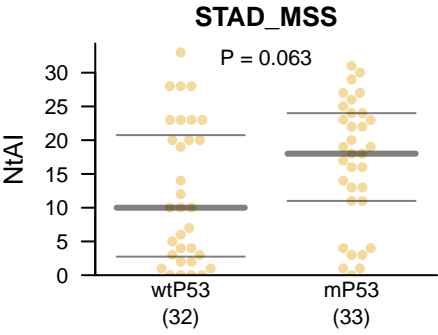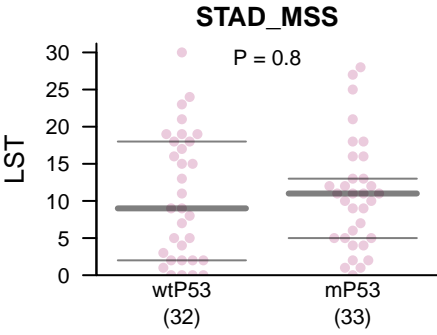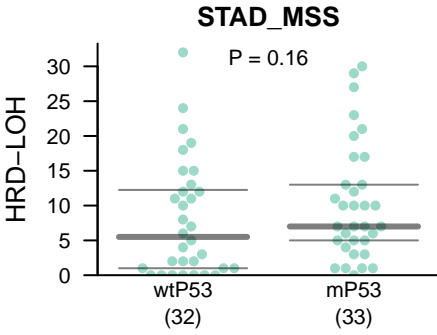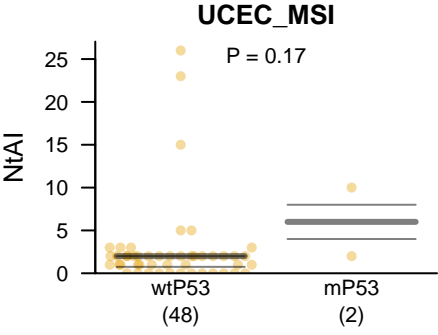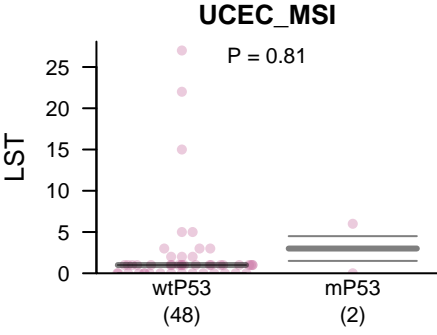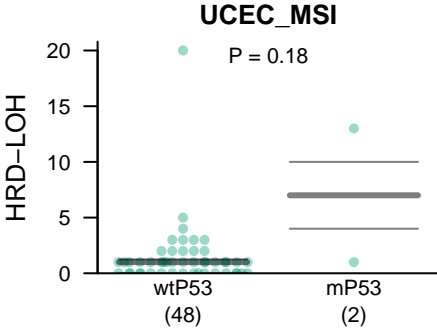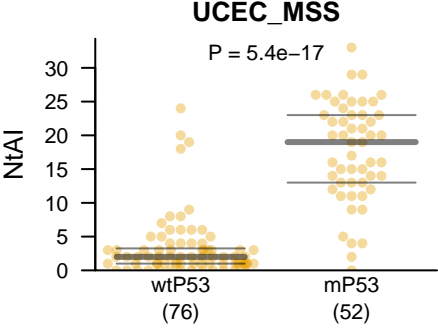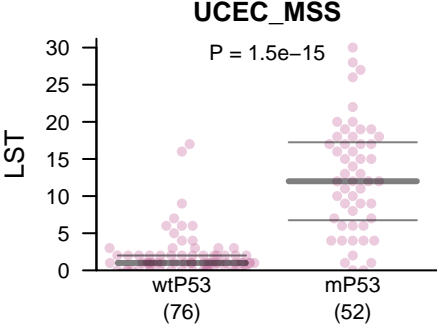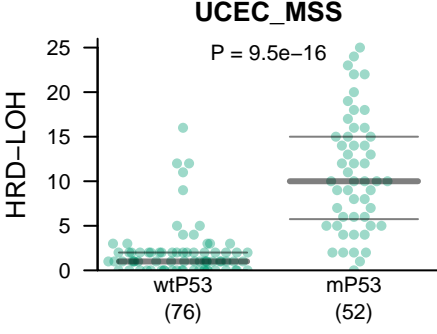

Supplement: Additional file 4: Figure S4. — Signature scores by p53. Distribution of signature scores in tumors with wildtype and mutant p53. [file 40364_2015_33_MOESM4_ESM.pdf]

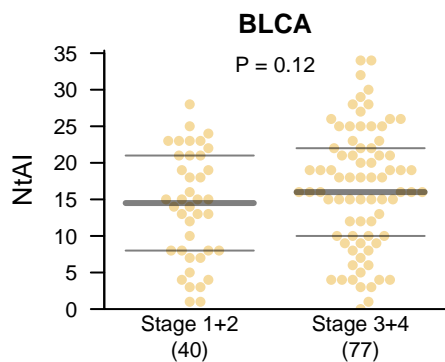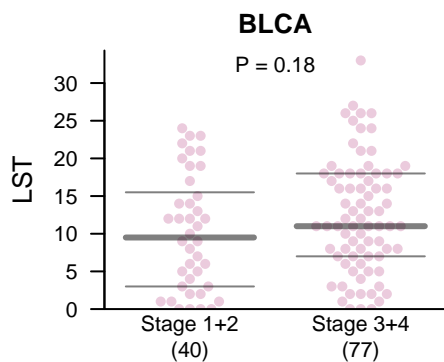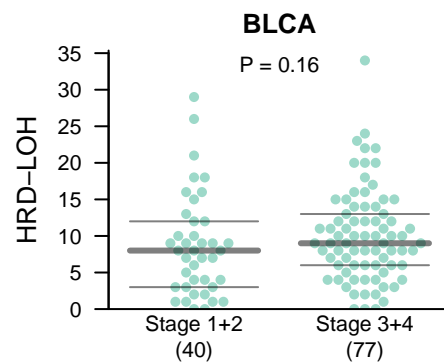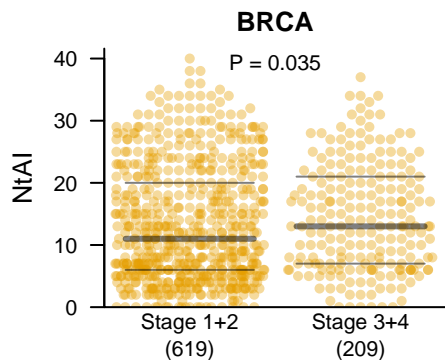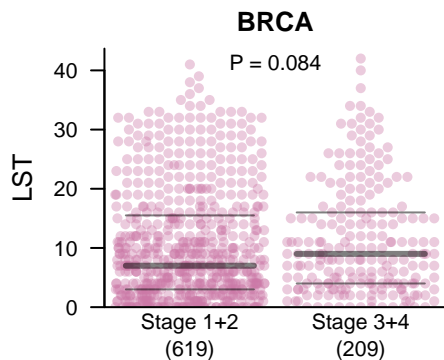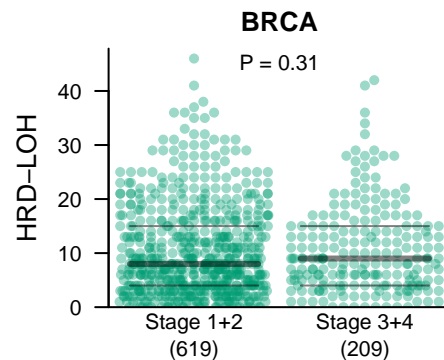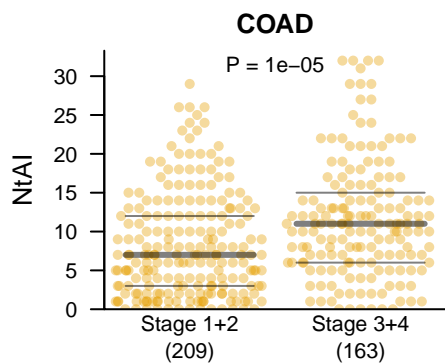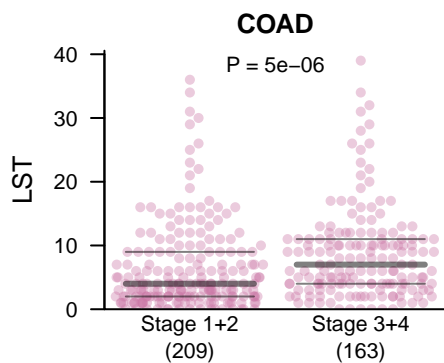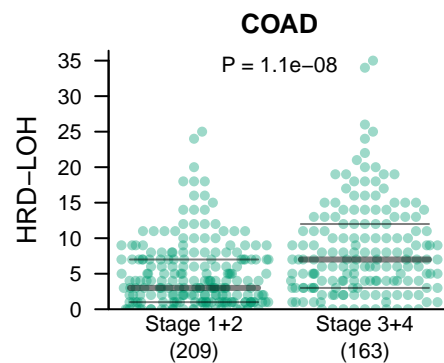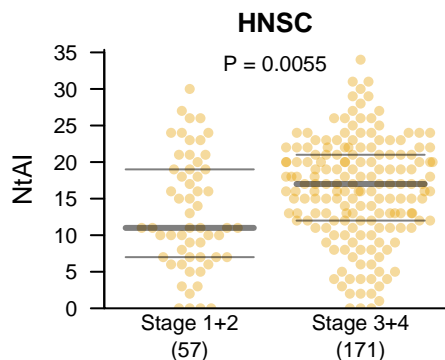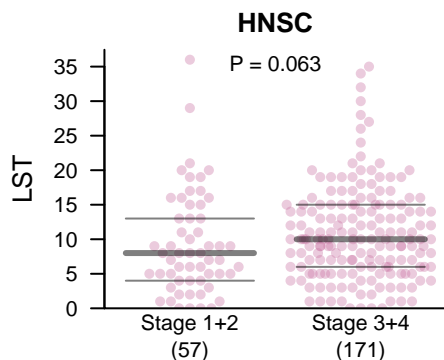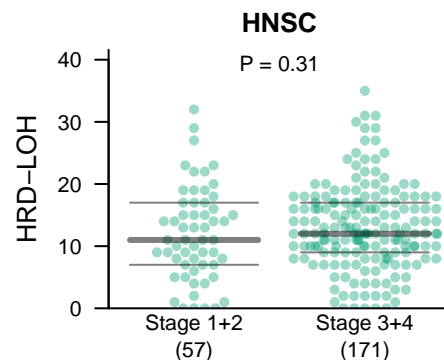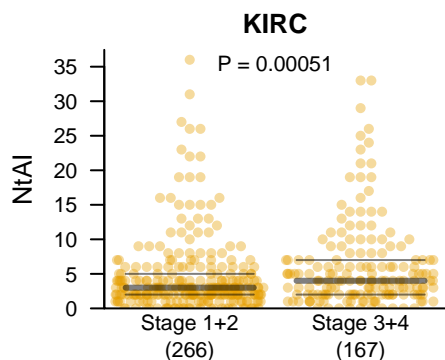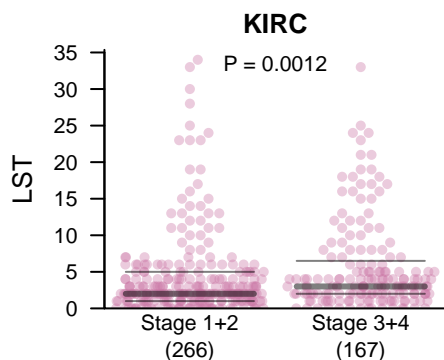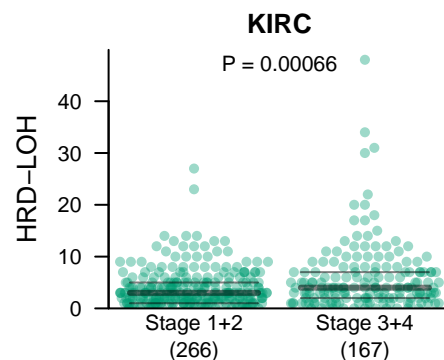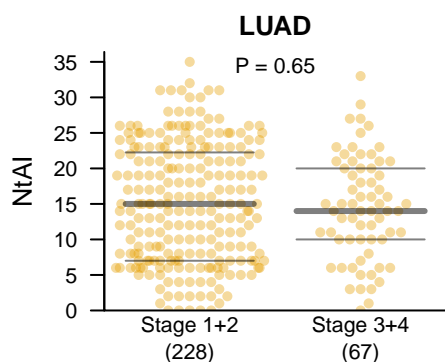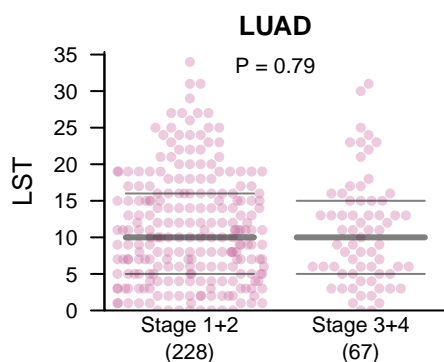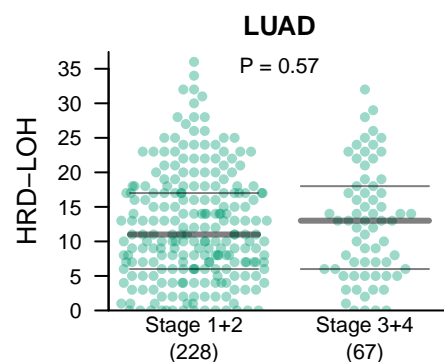

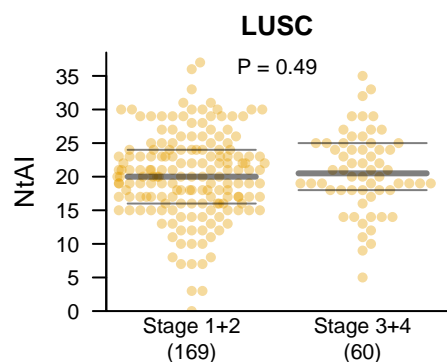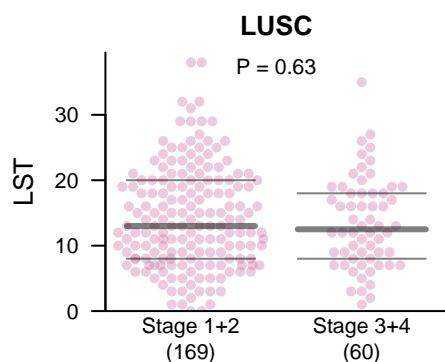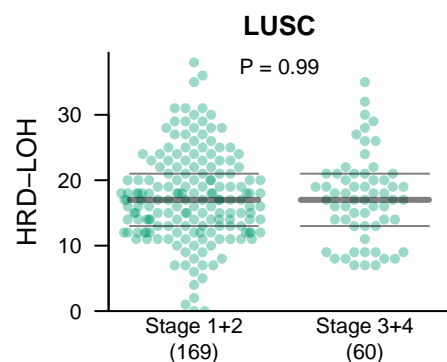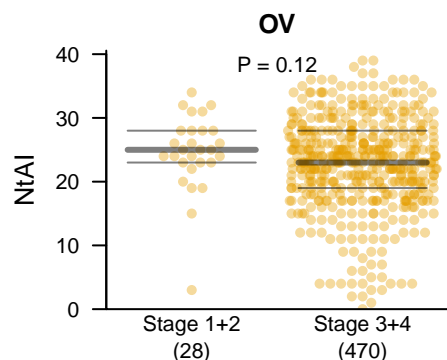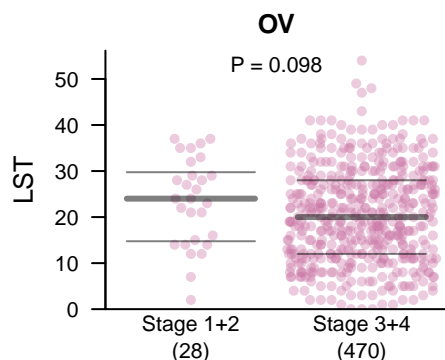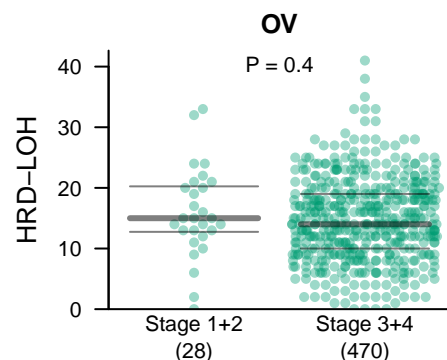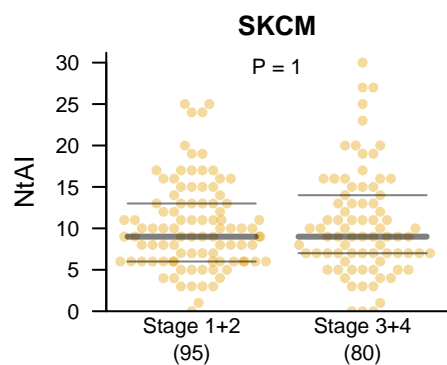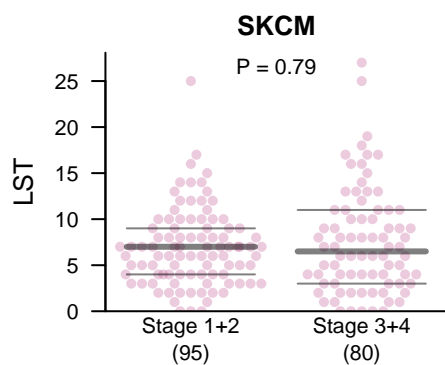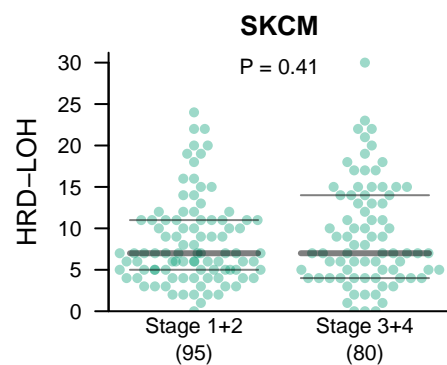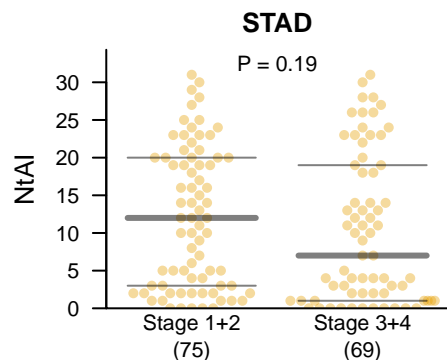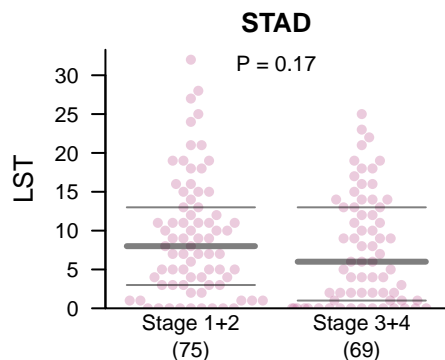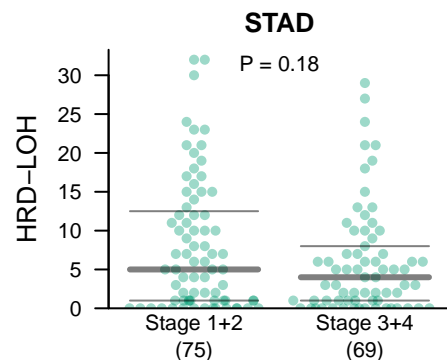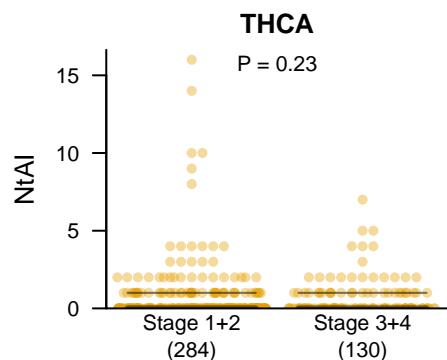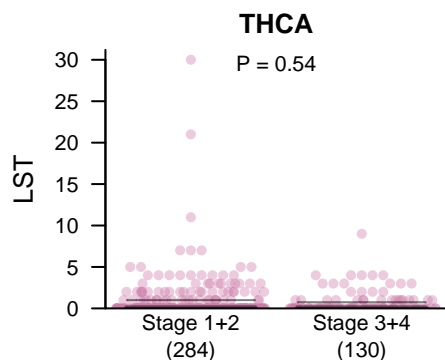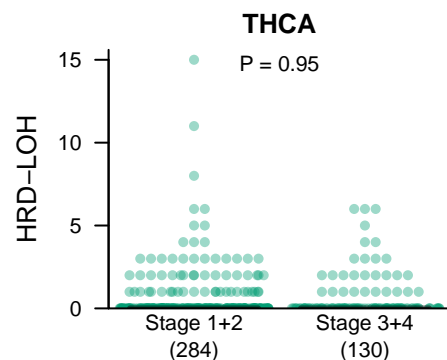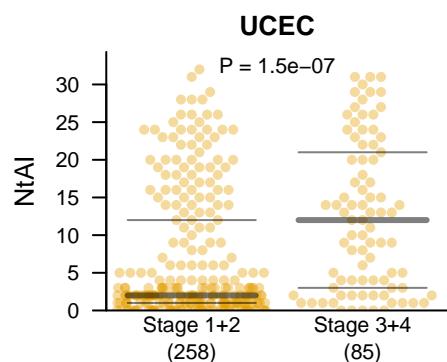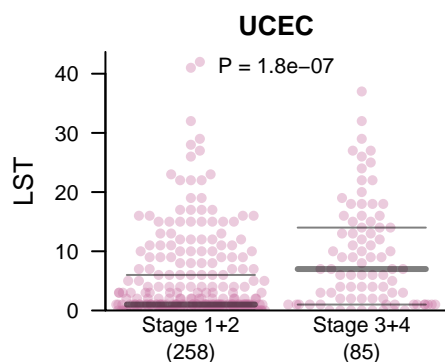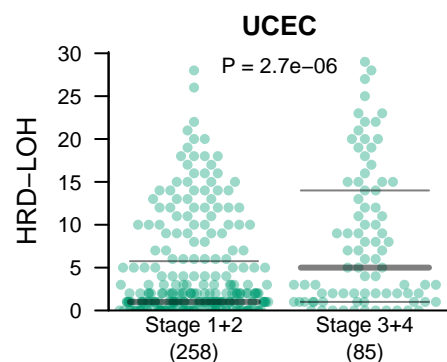

Supplement: Additional file 5: Figure S5. — Signature scores by stage. Distribution of signature scores by tumor stage. Low stage is defined as stage 1–2; high stage is defined as stage 3–4. [file 40364_2015_33_MOESM5_ESM.pdf]

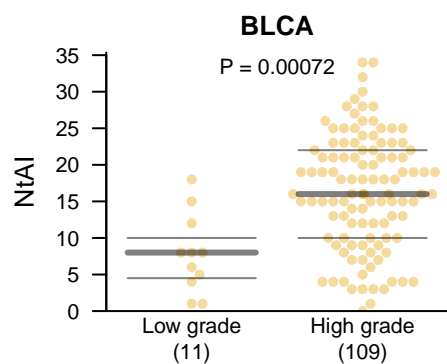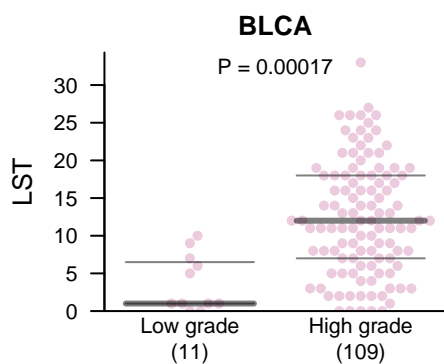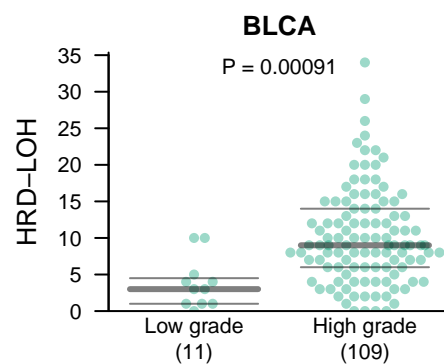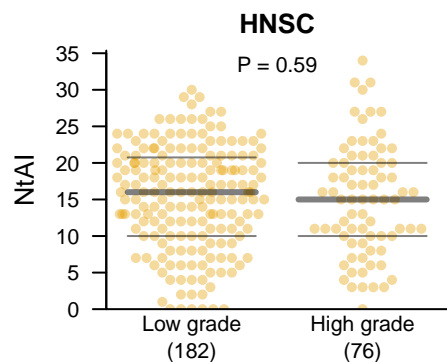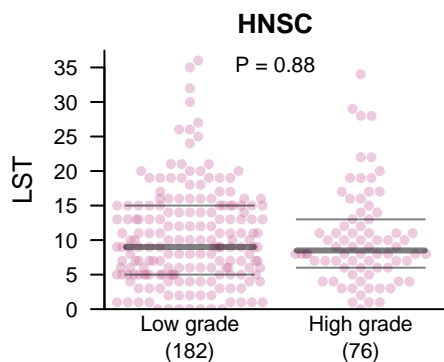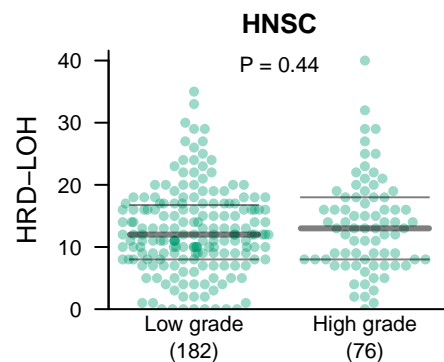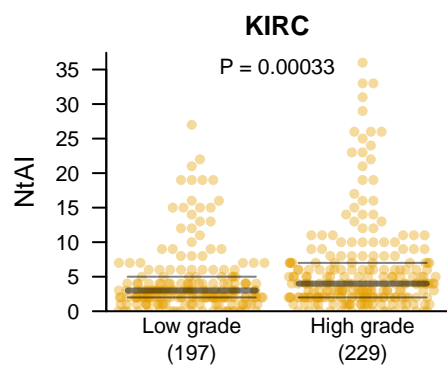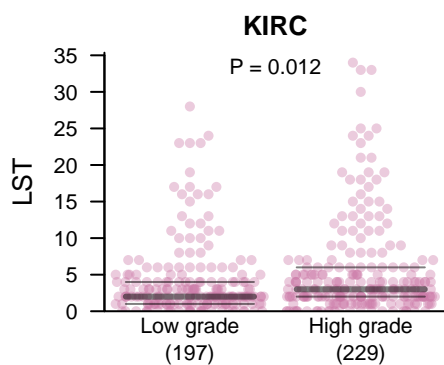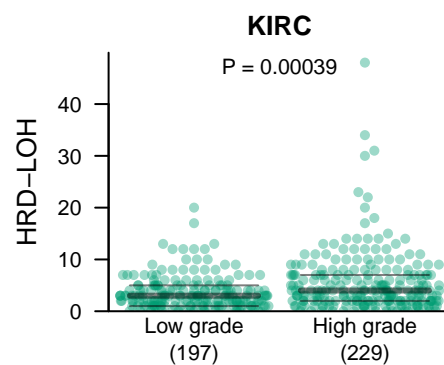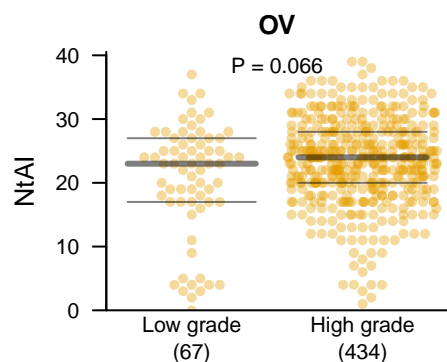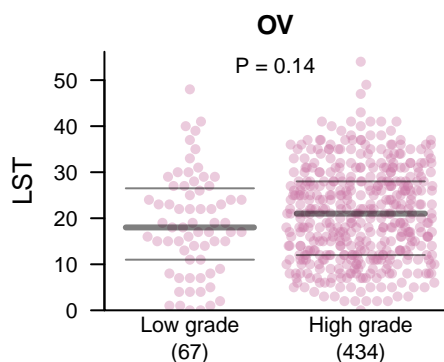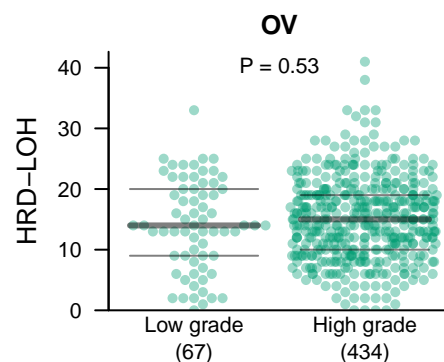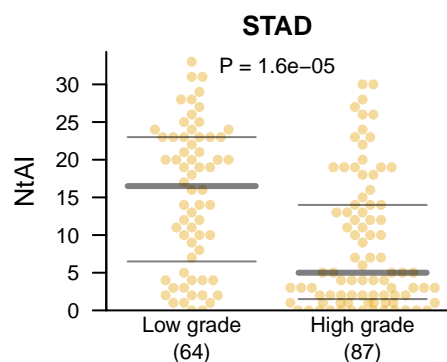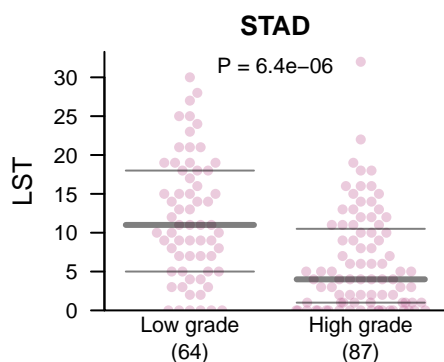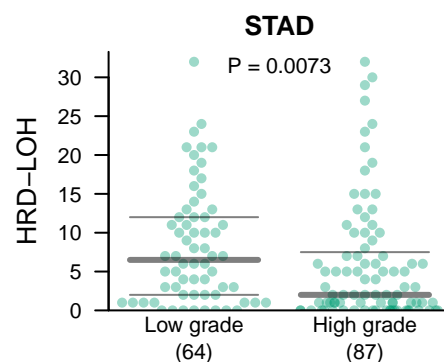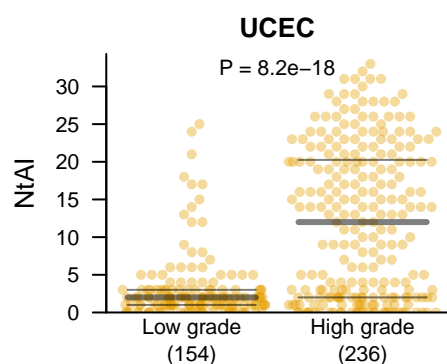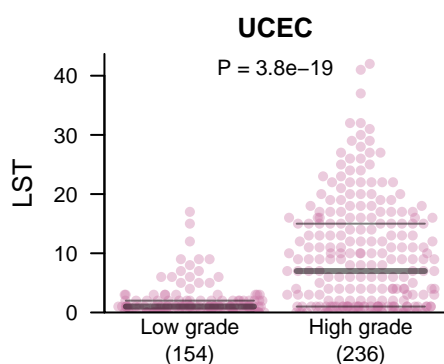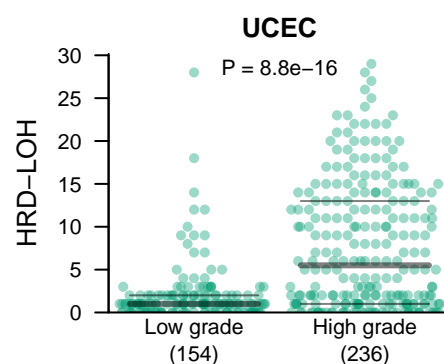

Supplement: Additional file 6: Figure S6. — Signature scores by grade. Distribution of signature scores by tumor grade. Low grade is defined as grade 1–2; high grade is defined as grade 3–4. [file 40364_2015_33_MOESM6_ESM.pdf]

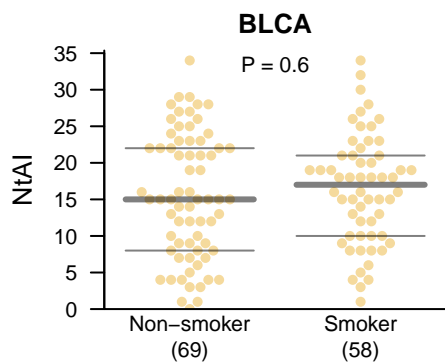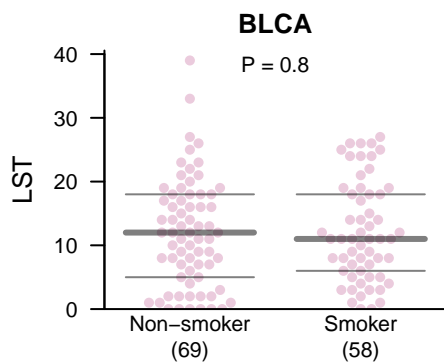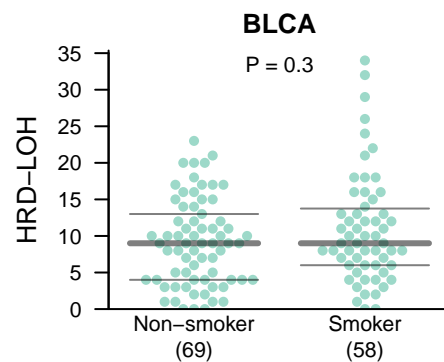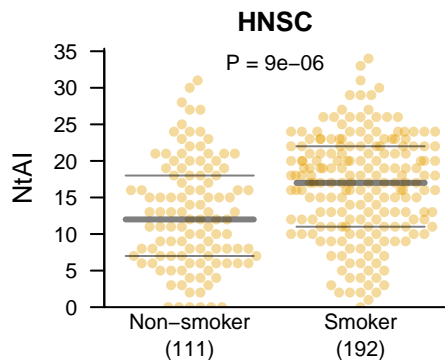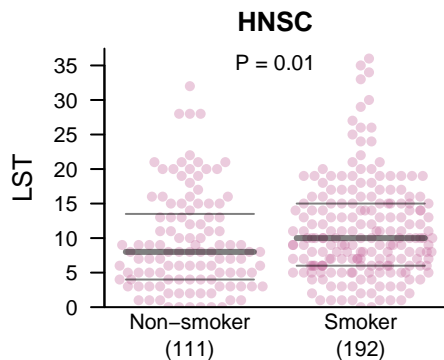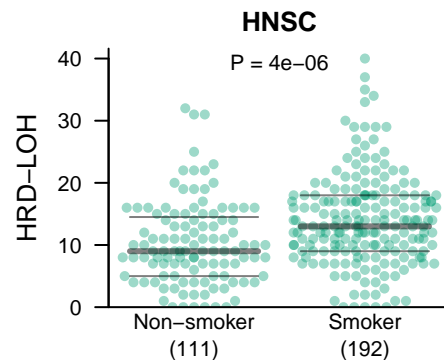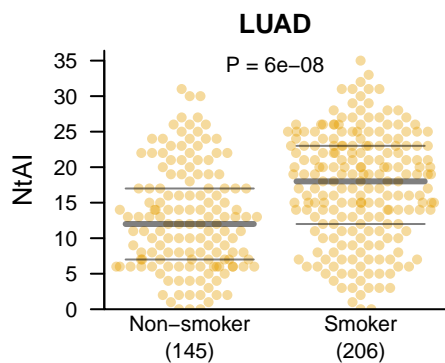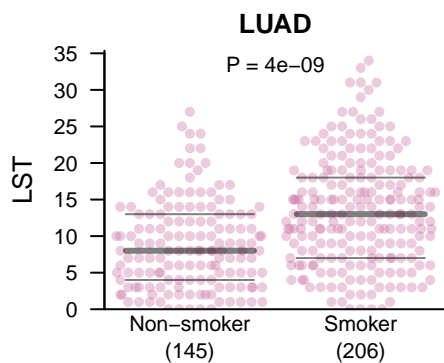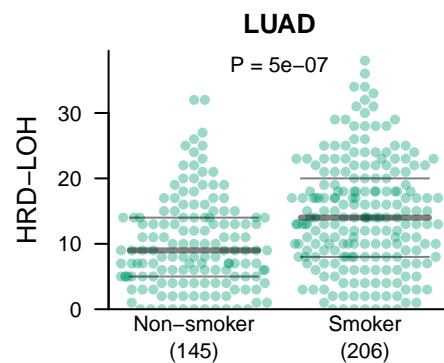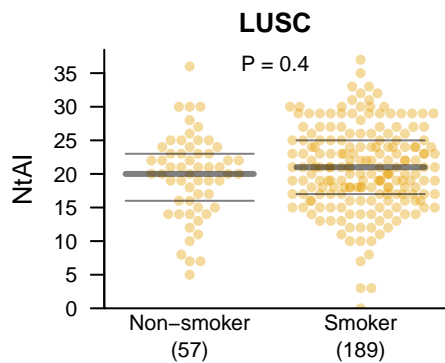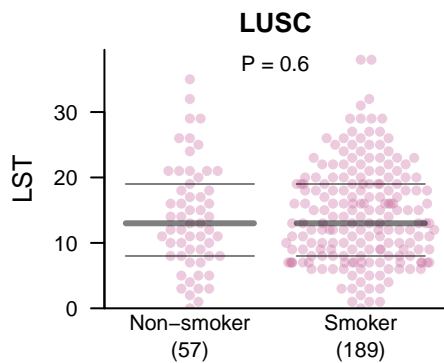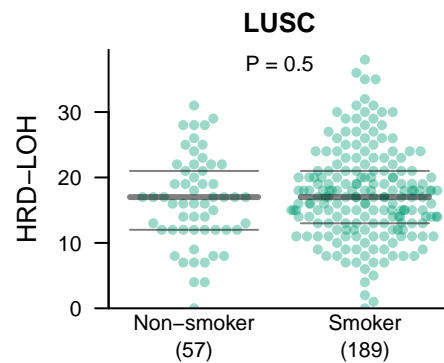

Supplement: Additional file 7: Figure S7. — Signature scores by smoking status. Distribution of signature scores by smoking status. A non-smoker is defined as a never-smoker or non-smoker for at least 15 years. A smoker is defined as a current or recent smoker (<15 years). [file 40364_2015_33_MOESM7_ESM.pdf]
